# Synthetic horizontal gene transfer for ecosystem restoration

**DOI:** 10.1186/s13062-026-00919-9

**Published:** 2026-08-14

**Authors:** Victor Maull, Guim Aguadé-Gorgorió, Victor de Lorenzo, Ricard Solé

**Affiliations:** 1 ICREA-Complex Systems Lab, University Pompeu Fabra, Dr Aiguader 80, Barcelona, 08003 Spain; 2 Institut de Biologia Evolutiva, CSIC-UPF, Pg Maritim de la Barceloneta 37, Barcelona, 08003 Spain; 3 ISEM, University Montpellier, CNRS, IRD, Montpellier, France; 4 Centro Nacional de Biotecnologia, CSIC, Madrid, Spain; 5 Santa Fe Institute, 1399 Hyde Park Road, Santa Fe, NM 87501 USA

**Keywords:** Synthetic biology, Ecological engineering, Climate change, Catastrophic shifts, Mutualism

## Abstract

Restoring endangered ecosystems has become a pressing issue as the effects of global warming continue to harm existing communities. During the last decade, several studies have suggested the use of synthetic biology as a tool to protect these biodiverse communities by increasing their functionality. A critical example concerns soil microbiome communities in drylands, where increasing water retention by some of the constituent species could effectively protect the ecosystem from abrupt degradation. However, how to effectively deploy a functional synthetic construct that can scale its impact to the community level remains an open question. Recent experimental research has designed recombinant gene plasmids with the capacity to horizontally transfer across soil microbial communities. Here, we explore the impacts of synthetic horizontal gene transfer in models of ecological consortia. We define a consumer-resource model in which species can share an engineered plasmid that reduces resource loss, effectively coupling multispecies dynamics and gene spreading. By doing so, we identify a wide range of parameters and the optimal gene transfer conditions for which the intervention promotes biodiversity and biomass gains, while regulating gene propagation and the spread of engineered organisms. Our work provides a first step toward understanding the mechanisms and opportunities of engineered plasmid transfer in multispecies ecological communities.

**Supplementary information:**

The online version contains supplementary material available at https://doi.org/10.1186/s13062-026-00919-9.

## Introduction

During the past two centuries since the Industrial Revolution, anthropogenic pressures, including accelerating climate change, pollution, intensive agriculture, grazing, and soil erosion, compounded by widespread habitat and biodiversity loss, have placed the stability of the planet in jeopardy. Many of these trends continue to accelerate, and models project multiple scenarios of ecosystem collapse in the coming decades [1–5]. In response, global efforts have increasingly focused on sustainable land and resource management, biodiversity conservation, renewable energy transitions, and sustainable development [6]. However, these measures alone may be insufficient, as multiple processes in the Earth’s system are rapidly approaching or exceeding the planetary boundaries that define a safe operating space for humanity [7].

**Fig. 1:**
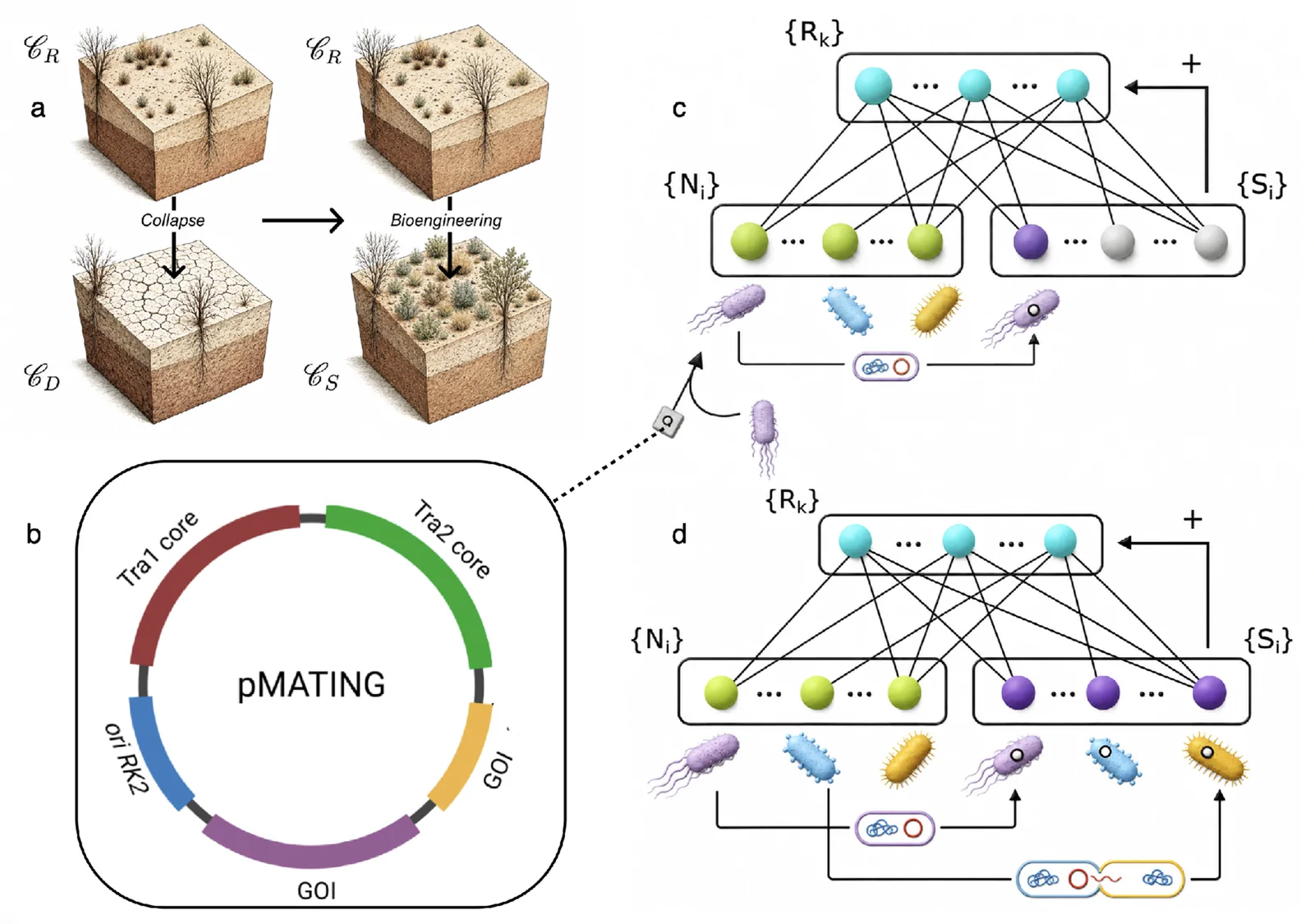
Engineering ecological networks using Horizontal Gene Transfer. (**a**) Ecosystem trajectories. An endangered ecosystem ($\mathscr{C}_R$) may either collapse ($\mathscr{C}_D$) or be shifted toward recovery ($\mathscr{C}_S$) through bioengineering. (**b**) The “pMATING” system enables propagation of recombinant genes via horizontal transfer in microbiomes. A gene of interest (GOI) is inserted into a bacterial host, which then spreads the plasmid via conjugation. (**c**) The model approach is based on a resource–consumer network model. Resources (${R_k}$) are consumed by native (${N_i}$) and synthetic (${S_i}$) plasmid carrier species. Initially, only the re-introduced host in (${S_i}$) carries the plasmid. (**d**) After *Δ t*, horizontal transfer occurs, leading to competition and GOI expression, which enhances resource stability. This mechanism could promote ecosystem recovery

Beyond mitigation and conservation, engineered intervention strategies have been proposed, such as geoengineering approaches for carbon removal or large-scale afforestation. These top-down methods offer more controllable results, but often come with high costs, scalability constraints, and uncertain ecological side effects [8]. In contrast, an alternative approach draws on the intrinsic capability of living systems themselves. Based on synthetic biology, this bottom-up restoration strategy proposes the design or modification of microbial communities to restore ecosystem functionality or confer resilience under environmental stress [9–15]. Examples include the restoration of coral reefs [16] and soils [17], the improvement of the dryland microbiota [18], and broader frameworks for ecological bioengineering [19]. Although diverse in mechanism and context, these strategies coalesce around a shared goal: employing biological agents to steer ecosystems back toward desirable, stable states or toward new, resilient configurations, see Fig. 1a. In this paper, we build on these concepts by advancing a synthetic biology-based intervention scenario in which ecosystem functions, rather than engineered organisms themselves, are propagated through native microbial communities using the naturally occurring process of horizontal gene transfer (HGT) as a delivery mechanism. We ask whether engineering such transmissible functions can improve ecosystem recovery and resilience while minimizing disruption of the original community structure.

The starting point is the identification of naturally occurring or laboratory-engineered biological materials -such as microbial strains or consortia- that possess superior catalytic abilities. These may range from the degradation of recalcitrant or toxic chemicals to fixation with $CO_{2}$ and $N_{2}$, to capture residual humidity or tolerating soil alkalinization after fires. In addition, modern genetic techniques make it possible to design and program microorganisms *à la carte*, combining traits within a single agent that are otherwise scattered in nature. This opens the door to an unprecedented repertoire of biological activities to mitigate the harmful impacts of urban and industrial processes. However, the challenge lies not only in discovering or enhancing (whether recombinantly or not) such active biological agents but also in developing effective strategies for their large-scale delivery and persistence. By analogy to the administration of drugs to the human body, this problem has been appropriately termed Environmental Galenics [19]. In other words, once we have an activity of environmental interest in hand, how can we deliver it extensively to target ecosystems safely and effectively?

A key challenge for all bioengineering strategies is ensuring that laboratory-made constructs spread effectively, ideally scaling with community size. In this respect, it is highly informative to leverage how microbial communities naturally acquire new biodegradation or resistance traits to cope with environmental stress [20]. Typically, this occurs when one member of the community evolves a solution to a specific challenge, and that DNA-encoded innovation is subsequently propagated, sometimes very rapidly, to neighboring organisms and other community members. Therefore, HGT involves all transfers of genetic material from one cell to another. HGT has traditionally been described through three distinct mechanisms: conjugation, transformation, and transduction. Conjugation, in particular, requires direct contact between donor and recipient cells via a pilus, through which genetic material is transferred, and appears to be the most promising short-term strategy for DNA transfer to a wide range of potential bacterial recipients [19, 21, 22]. This principle is particularly evident in the spread of antibiotic resistance. Although this phenomenon raises major concerns in the biomedical field, it also reveals the existence of highly efficient DNA transmission channels that enable the rapid dissemination of beneficial traits across communities.

Unfortunately, the strong association between HGT and antibiotic resistance has often obscured the enormous opportunity to repurpose these very same mechanisms for positive outcomes. By harnessing conjugation and related processes through synthetic biology, it becomes possible to deliberately propagate advantageous activities; a kind of targeted enhancement of the catalytic capacities of microbes within a given ecosystem. This is particularly relevant in the context of the central role that microbial diversity plays in a wide range of ecological scenarios [23]. This includes diversity as a barrier against invaders and pathogens [16, 24, 25], as a driver of multifunctionality [26], climate mitigation [27], and as an intrinsic component of engineering strategies where it acts as a firewall [28].

In this context, the work of Aparicio et al. (2022) is especially noteworthy [29]. This work has experimentally demonstrated the feasibility of engineering hyperpromiscuous plasmids (Fig. 1b) in laboratory donor strains, enabling the in situ transfer of combinatorial gene sets to diverse soil bacteria. Although the study was limited to a proof-of-concept model system, the results strongly suggest that scaling up this strategy with vectors carrying functions of environmental relevance could provide powerful tools for restoring ecosystems damaged by chemical pollutants and atmospheric stressors. Could these engineering approaches be used to restore ecosystems?

In order to address the previous questions, mathematical modeling efforts can help guide experimental design and deepen mechanistic understanding of ecological tipping points and the engineering to restore [30, 31]. In addition, modeling diverse communities can help predict the potential outcomes of HGT-based intervention scenarios. This paper aims to support such approaches by proposing a minimal model that incorporates three key ecological features, namely:

1. Multi-species interactions, to reflect community-level dynamics and account for the role of biodiversity in resisting ecosystem collapse and providing ecosystem services and reliability through functional redundancy [24, 26, 32, 33].
2. Environment-community feedbacks, with niche construction [34] as a central concept, modeled using extensions of the Lotka-Volterra equations in which resource dynamics are considered explicitly [35, 36].
3. Gene spread as an epidemiological process, building upon classical theoretical models of horizontal gene transfer and plasmid dynamics [37–43], reviewed in [44]. More recently, these ideas have been extended to show how HGT can promote microbial coexistence and diversity [45]. Unlike these studies, which primarily address plasmid persistence, evolutionary dynamics, or community coexistence, our focus is on the dissemination of engineered ecological functions and their ecosystem-level consequences.

This approach enables the integration of desired functions at the multispecies level, overcoming the limitations of single-species engineering. This paper aims to examine the potential effects and robustness of this strategy. As our model shows, HGT spreading of a functionally beneficial construct can enhance both the efficiency of the intervention and its community-level impacts while having no cascade effects nor other unintended consequences.

## Methods

The backbone of our model is a two-layered network *ξ* of interaction between the extant species of a given community and the available resources, see Fig. 1c. In parallel, a potential, yet non-existent, synthetic community shares the same *ξ* interaction context. The first synthetic resident would be introduced through an initial anthropogenic action: the addition of a synthetic species, which essentially comprises a modified native species that harbors a highly transmissible plasmid, i. e. plasmid pMATING, described by [29], see Fig. 1b. Once the first synthetic is introduced, the propagation of the recombinant genes will spread across the extant community by HGT, see Fig. 1d. Interestingly, the synthetic genetic material accommodates a gene of interest section (GOI) for delivering heterologous genes to wild-type mating partners. Such GOI can potentially induce specific functionalities that would benefit the endangered community.

In a previous work [28], we studied how slowing down the depletion of shared resources leads to a rise in biomass and biodiversity. Additionally, we highlight the seamless integration of synthetic species into the ecological network, accompanied by minor yet positive shifts in the population size of the existing community. One particular scenario is outlined in [28]. A synthetic species capable of producing an exopolymer could help mitigate water loss, thereby positively influencing the other community members. For example, the production of a simple exopolymer, such as hyaluronic acid [46, 47], might involve a cassette of genes encoded within the gene of interest (GOI) section of a pMATING plasmid.

**Fig. 2:**
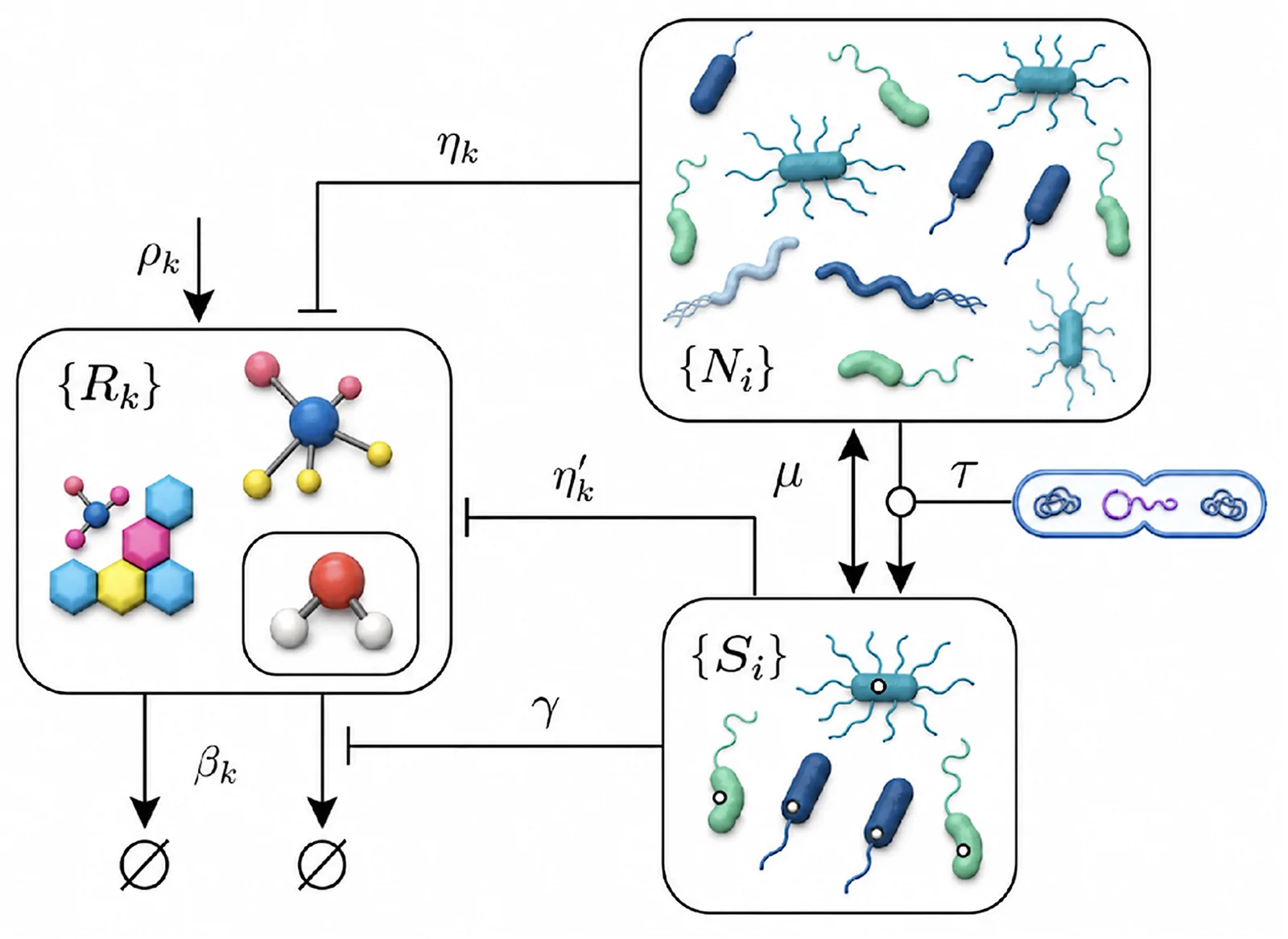
The multi-resource, multi-species framework. The extant community resides the upper box as $\{N_i\}$, growing while consuming resources from the left compartment $\{R_k\}$ at a given rate. Upon the introduction of the first synthetic inoculum carrying the plasmid (white circle), horizontal gene transfer (HGT) enables plasmid transmission across the community, causing a fraction of species to transition into the synthetic compartment $\{S_i\}$ at a rate *τ*. These synthetic species also consume resources from the same pool. Plasmid loss occurs at a rate *μ*, leaking back to $\{N_i\}$. Additionally, the synthetic community $\{S_i\}$ affects $\{R_k\}$ decay rate $\beta_k$

We tackle mathematically this problem by building a system of coupled ordinary differential equations describing (1) inorganic resource availability, denoted by $R_k$, where *k = 1,2,…,m*, (2) a set of resident species, denoted by $N_i$, where *i = 1,2,…,n*, and (3) a corresponding set of plasmid-bearing strains, denoted by $S_i$, with one potential plasmid-bearing counterpart associated with each resident species. The resulting dynamical system therefore contains *m* resource variables and *2n* population variables, for a total of *m+2n* coupled ordinary differential equations. For clarity, a diagram of the model is shown in Fig. 2.

1 $${d R_{k} \over dt} = \rho_{k} - \underbrace{\left (\sum_{i}^n \xi_{k i} N_{i} \right ) R_{k}}_{\text{R consumed by N}} - \underbrace{\left (\sum_{j}^n \xi_{k j} S_{j} \right ) R_{k}}_{\text{R consumed by S}} - \underbrace{F_k(R_{k},\mathbf{S})}_{\mathrm{Decay}}$$

2 $${d N_i \over dt} = \underbrace{\eta_i N_{i} \left (\sum_{k}^m \xi_{i k} R_{k} \right )}_{\text{N consumes R}} - \underbrace{\tau N_{i} \sum_{j}^n S_j}_{\mathrm{HGT}} + \underbrace{\mu S_i}_{\text{Plasmid loss}} - \underbrace{\sigma_i N_i}_{\mathrm{Decay}} + \underbrace{\epsilon}_{\mathrm{Migration}}$$

3 $${d S_i \over dt} = \underbrace{\eta ^{\prime}_{i} S_{i} \left (\sum_{k}^m \xi_{i k} R_{k} \right )}_{\text{S consumes R}} + \underbrace{\tau N_{i} \sum_{j}^n S_j}_{\mathrm{HGT}} - \underbrace{\mu S_i}_{\text{Plasmid loss}} - \underbrace{\sigma_i S_i}_{\mathrm{Decay}}$$

The resources equation has four main parts. The intrinsic, resource-dependent growth rate is represented by $\rho_k \in [0,1]$, the consumption term by $N_{i}$, the consumption term by $S_{i}$, and a decay term $F_k(R_{k}, \mathbf{S})$ that entangles the synthetic functionality within which the mechanism is detailed separately below.

The equation of extant species has five main parts. The growth rate $\eta_{i} \in [0,1]$ as a classic replicator, dependent on $R_{k}$ consumption by means of $\xi_{ik}$ that weighs the strength of the resource-consumer interaction. The terms in *ξ* come from a uniform rectangular distribution U [0, 1], with connectivity *C=0.3*. The HGT term depends on the transmissibility *τ* of the recombinant vector and the peer-to-peer encounter with a conjugated synthetic donor. Plasmid loss is allowed, therefore $S_i$ is transformed to $N_i$ at rate *μ*. Finally, there is a decay rate, represented by $\sigma_{i} \in [0,1]$, and to prevent unrealistically low biomass, a small immigration factor of $\epsilon = 1 \times 10^{-5}$ is introduced.

The synthetic strain grows at a rate $\eta^{\prime}_i \in [0,1]$, which is assumed to be lower than that of their wild-type counterparts, i.e., $\eta^{\prime}_i < \eta_i$, due to the metabolic burden imposed by the recombinant load. The resource-consumer interaction matrix $\xi_{ik}$ is shared between both wild-type and synthetic species. The HGT term remains the same but contributes positively, since it represents the recruitment of individuals carrying plasmids. In contrast, *μ* denotes the plasmid loss rate. The decay rate $\sigma_i$ is assumed to be unchanged. Note that there is no immigration term for the synthetic population. While the wild-type community benefits from an established ecological assembly process (with a rate immigration term *ε*) the synthetic population $S_i$ originates solely through HGT, at least during the initial phase of ecological engineering.

Finally, the effect encoded in the plasmid is captured by the following function:

4 $$F_k(R_k,\mathbf{S}) = \frac{\beta_k R_k}{1+\delta_k\displaystyle\frac{\sum_{i=1}^{n}S_i}{\gamma}}$$

5 $$ \delta_k = \begin{cases} 1 & \text{if resource }k\text{is targeted by the plasmid},\\0 & \mathrm{otherwise}.\end{cases}$$

The function $F_k(R_k,\mathbf{S})$ introduces a non-linear adjustment to the resource decay dynamics, where $\mathbf{S}=(S_1,\ldots,S_n)$ denotes the vector of plasmid-bearing species abundances. The saturating form of this function was chosen to represent an ecosystem engineering process with finite capacity, whereby additional synthetic biomass yields progressively smaller reductions in resource degradation. Similar inhibitory responses are widely used in systems biology and regulatory network models to describe negative feedback processes and saturating control mechanisms [48]. Specifically, the existence of synthetic species in the community induces a damping effect on the degradation of a specific resource or a group of target resources. The maximum decay rate for a given resource *k* is denoted by $\beta \in [0,1]$, while the constant $\gamma \in [0,1]$ represents the rate at which the degradation process (*β*) is inhibited due to the presence of synthetic strains. Hence, the damping effect exclusively influences the targeted resources, i.e., those for which $\delta_k=1$. Any subset of the resources $\{R_k\}_{k=1}^{m}$ can be targeted, ranging from a single resource to the entire resource pool.

### Model extensions and robustness analyses

The model described above assumes that plasmid-bearing and plasmid-free strains share identical ecological interaction profiles and that horizontal gene transfer is governed by a homogeneous conjugation parameter *τ.* To evaluate the robustness of the model predictions, we systematically relax these assumptions and introduce heterogeneity in both ecological interactions and conjugation abilities.

First, we allow plasmid carriage to modify resource-consumption preferences by assigning plasmid-bearing strains a resource-consumption matrix $\xi_S$ distinct from the wild-type matrix $\xi_W$. The degree of divergence between both matrices was controlled by a heterogeneity parameter *h*, such that

6 $$\xi_{S,ij} = \xi_{W,ij}\left(1+\zeta_{ij}\right),$$

where $\zeta_{ij} \sim U[-h,h]$. This procedure generates increasing ecological differentiation between plasmid-free and plasmid-bearing strains while preserving the overall topology of the original resource–consumer interaction network.

Second, we replace the homogeneous transfer parameter *τ* with species-specific donor ($d_i$) and recipient ($r_i$) abilities. Horizontal gene transfer is then modeled as

7 $$\mathrm{HGT}_i = r_i N_i \sum_{j=1}^{n} d_j S_j,$$

where donor and recipient abilities are obtained from correlated lognormal distributions. To generate the desired correlation structure, we first draw a standard Gaussian random variable $x_i\sim\mathscr{N}(0,1)$ for each species and construct a second Gaussian variable according to

8 $$y_i=c_{DR}x_i+\sqrt{1-c_{DR}^2}\,z_i,$$

where $z_i\sim\mathscr{N}(0,1)$ is an independent Gaussian random variable. Donor and recipient abilities are then obtained by exponentiating $x_i$ and $y_i$, respectively. Thus, the parameter $c_{DR}$ controls the association between donor and recipient abilities, while preserving the same average conjugation scale across all scenarios. The original homogeneous-transfer model is recovered when all species have identical donor and recipient abilities, $d_i=r_i=\sqrt{\theta}$, yielding

9 $$\mathrm{HGT}_i = \theta N_i \sum_{j=1}^{n} S_j,$$

where *θ* is directly comparable to the original homogeneous transfer parameter *τ*.

Positive values of $c_{DR}$ produce communities in which efficient donors also tend to be efficient recipients (superspreaders), whereas negative values generate a trade-off between donating and receiving abilities. We considered three representative scenarios corresponding to positive ($c_{DR}=1$), null ($c_{DR}=0$), and negative ($c_{DR}=-1$) donor–recipient correlations.

Finally, to evaluate the sensitivity of the results to the functional form of ecosystem engineering, we replaced the saturating resource-modification function Eq. 4, with the capped linear formulation

$$F_k(R_k,\mathbf{S})= \beta_kR_k \max\left(0,\,1-\delta_k\frac{\sum_iS_i}{\gamma}\right),$$

while leaving all other model components unchanged.

## Results

### Low-dimensional model: predictive insights

**Fig. 3:**
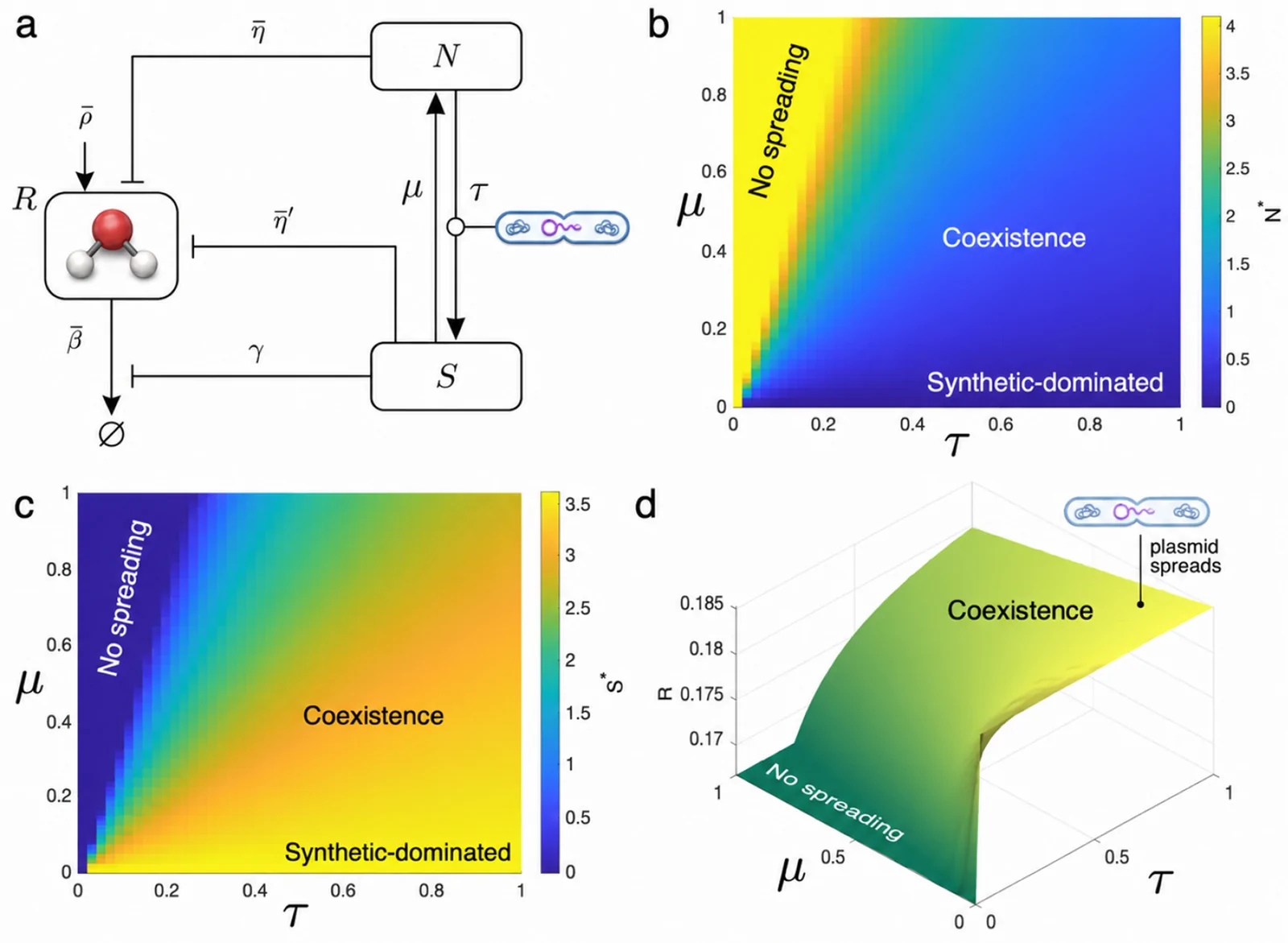
A coarse-grained, low-dimensional model of HGT dynamics. Here, the diverse community structure is reduced to pairwise dynamics (Eqs. 17–19) between a wild-type species *N* and its synthetic counterpart *S*, both competing for a single resource *R*. The results from this model (see SM for details) are shown in (**b-d**), where we plot the stable populations of (**b**) wild type, (**c**) synthetic, and (**d**) resource levels. Two well-defined phases exist, associated with the failure of transmission and its success. Here (**a**): *ρ = 0.8*, *β = 0.7*, *γ = 0.3*, *η = 0.6*, $\eta^{\prime} = \eta - 0.1\eta$ and *σ=0.1*

As an initial approach, we analyzed a simplified coarse-grained resource-consumer system Fig. 3a. In this way, we can obtain some relevant insight concerning the expected behaviour of our multidimensional system. One way of coarse-graining the original model is to assume a homogeneous set of parameters, namely that all parameters can be well represented by some average, constant value. In our case that means taking $\sigma_i=\sigma$, *ε = 0*, and $\xi_{ij}=\xi$. Additionally, without loss of generality, we take *ξ=1*. The total values of the variables can be represented as $R = \sum_{k} R_k$, $N = \sum_i N_i$ and $S = \sum_i S_i$, therefore we study the coarse-grained dynamics described by:

10 $${d R \over dt} = {\sum_{k}^{m}\rho_k - N R - S R - \sum_{k}^{m} F_k(R_k,S)}$$

11 $${d N \over dt} = {R \sum_{i}^{n}\eta_i N_i - \tau S N + \mu S - \sigma N}$$

12 $${d S \over dt} = {R \sum_{i}^{n}\eta^{\prime}_i S_i + \tau S N -\mu S - \sigma S}$$

The function $\sum_{k=1}^{m} F_k(R_k,S)$ stands for

13 $$\sum_{k=1}^{m} F_k(R_k,S)=\sum_{k=1}^{m}\frac{\beta_k R_k}{1+\delta_k\frac{S}{\gamma}}.$$

Assuming that all resources are affected by the synthetic functionality, we have $\delta_k=1$ for all *k*. The inhibition term therefore becomes identical for all resources and can be factored out of the summation, meaning that the coarse-grained synthetic population reduces resource loss as

14 $$\sum_{k=1}^{m} F_k(R_k,S)=\frac{\sum_{k=1}^{m}\beta_kR_k}{1+\frac{S}{\gamma}}.$$

For the rest of the parameters, we use effective mean-field averages, assuming that species-specific deviations around the mean can be neglected for analytical tractability:

15 $$\bar{\rho} = \frac{\sum_k^{m} \rho_k}{m} \qquad \bar{\beta} = \frac{\sum_k^{m} \beta_k R_k}{R} $$

16 $$\bar{\eta} = \frac{\sum_i^{n} \eta N_i}{N} \qquad \bar{\eta}^{\prime} = \frac{\sum_i^{n} \eta^{\prime} S_i}{S}$$

Although $\bar{\eta}$ and $\bar{\eta}^{\prime}$ are defined as weighted averages, in the coarse-grained model they are subsequently treated as effective constant parameters, corresponding to a mean-field approximation in which all species are represented by typical growth rates.

Therefore, it can be shown that the previous Eqs. (1, 2, and 3) collapse in a three-dimensional model, namely:

17 $${d R \over dt} = {m\bar{\rho} - RN - RS- {{\bar{\beta} R} \over {1+{{S} \over {\gamma}}}}}$$

18 $${d N \over dt} = {\bar{\eta} NR - \tau NS + \mu S - \sigma N}$$

19 $${d S \over dt} = {\bar{\eta}^{\prime} SR + \tau NS -\mu S - \sigma S}$$

The analysis of this system reveals three fixed points, obtained from *dN/dt=dS/dt=dR/dt=0*, namely: (1) resource-only, $P_0=(R^*_0,0,0)$, (2) resource and wild type, $P_1=(R^*_1,N^*_1,0)$ and (3) the coexistence point, $P_c=(R^*_c,N^*_c,S^*_c)$. Here we define:

20 $$R^*_0 = {m \rho \over \beta} \;\; ;\;\; R^*_1 = {\sigma \over \eta} \;\; ; \;\; N^*_1 = {m \rho \eta\over \sigma}-\beta $$

An interesting first result is that none of the attractors contains $N^*=0$ and $S^* > 0$. This means that, in the mean field approximation, the mutant cannot completely overtake its wild-type counterpart, except for the limit case scenario where *μ = 0*. This shows that natural loss of the engineered plasmid by the synthetic strain is not detrimental but is, in fact, useful to keep the wild-type strain in place, suggesting that controlled plasmid instability may be a desirable design feature rather than a limitation.

Furthermore, the stability analysis of the coexistence phase shows that there is a critical boundary separating a domain of successful HGT (when $P_c$ is stable) from the extinction of the synthetic (when $P_1$ is stable) (see SM). This condition is a linear relation:

21 $$\mu_c(\tau)=\left(\frac{\eta^{\prime}}{\eta}-1\right) \sigma+ N_1^* \tau.$$

For $\mu < \mu_c(\tau)$, *S* propagates successfully and for $\mu > \mu_c(\tau)$, *S* cannot persist (see SM for details).

The basic properties and steady states of this system are summarized in the bifurcation diagrams shown in Fig. 3. Here we can see the equilibrium points for $N^*$, $S^*$, and $R^*$, against different values of two key parameters relevant for our engineering approach, namely the efficiency of the HGT process (*τ*) and the reversal of the process due to loss of plasmids, as weighted by *μ*. As discussed above, the system has two nontrivial stable attractors, involving either HGT failure (only the wild-type survives) or success, whose parameter domains are indicated in Fig. 3b-d as “no spreading” and “coexistence”. As predicted by the theory, a straight line $\mu_c(\tau)$ separates them. Interestingly, the region where HGT succeeds does not depend on the engineered function *F*: To ensure that a plasmid and synthetic strain *S* are maintained in a population, we only need to focus on tuning *μ* and *τ* accordingly, consistent with classical theory on plasmid dynamics [37].

In that regard, and because the implemented function *F* reduces the degradation of the resource, we can see that higher *τ* implies higher *S* and, in turn, more resource (Fig. 3d). However, because this effect saturates at a certain *S*, the model indicates that we do not need to aim towards maximum *S*. Instead, a small presence of *S* can already increase the amount of resource while maintaining the wild-type strain in place (Fig. 3d). This again indicates that moderate values of the spread (*τ*) and loss (*μ*) of the plasmid might be sufficient to achieve the desired effect.

The main message from the low-dimensional model is that, provided that the rate of HGT is high enough to compensate for the plasmid loss rate, we should expect to observe a successful propagation of our engineered plasmid. In this coexistence phase, the engineered functionality would be effective without the need of a fully-synthetic community. We next ask if these predictions for co-existence and successful functionality at intermediate *τ* and *μ* values hold against a more realistic scenario, where multiple species and resources are explicitly introduced.

### Multispecies ecosystem

Using Eqs. (1)-(3), we numerically integrated a high-dimensional consumer–resource system with a fourth-order Runge–Kutta scheme. Each community had *n=20* species and *m=20* resources and was evolved to stability over *Δ t=200*. At $T_1$, we introduced an invading synthetic strain $S_i$ derived from a randomly chosen wild type $N_i$ with $N_i(T_1) > 0.2$, using an inoculum $S_i(T_1)=0.01$. The system was then simulated for 200 additional steps to a new equilibrium at $T_2$, allowing up to 20 synthetic counterparts (one per wild type) via HGT. Synthetic strains shared the wild-type interaction matrix, while plasmid acquisition imposed a *10%* metabolic burden, $\eta^{\prime}=0.9\,\eta$.

We swept plasmid transmissibility and loss rate, $\tau\in[0,0.15]$ and $\mu\in[0,0.3]$. For each $(\tau,\mu)$ pair, 200 independent replicas were run with randomized parameters $\rho_k\in[0.8,1]$, $\beta_k\in[0.7,0.9]$, *γ=0.3*, $\eta_i\in[0.5,1]$, $\sigma_i\in[0.5,0.7]$ and $\epsilon = 10^{-5}$. The resources affected by *S* are determined by $\delta_k$, see methods. Yet the results were shown to be robust across all parameter space, see SM.

Community change from $T_1$ to $T_2$ was quantified by: (i) synthetic relative abundance,

$$\mathrm{SRA} = 100 \times \frac{\sum S_{\mathrm{total}}}{\sum (S_{\mathrm{total}} + N_{\mathrm{total}})},$$

computed over species with abundance * > ε* and averaged over the 200 replicas per $(\tau,\mu)$; (ii) strain biodiversity fold change,

$$\mathscr{F} = \log_2\!\left(\frac{D(T_2)}{D(T_1)}\right),$$

with *D(t)* the number of strains (wild-type + synthetic) above *ε*; and (iii) biomass fold change,

$$\mathscr{G} = \log_2\!\left[\frac{\sum_{i=1}^{m}\big(N_i(T_2)+S_i(T_2)\big)}{\sum_{i=1}^{m}\big(N_i(T_1)+S_i(T_1)\big)}\right];$$

We report fold change on a $\log_2$ scale. Values * > 0* indicate an increase, * < 0* a decrease, and *0* no change. Key benchmarks: $\mathscr{F} | \mathscr{G}=1$ (doubling, *2×*), $\mathscr{F} | \mathscr{G}=-1$ (halving, *1/2×*), $\mathscr{F} | \mathscr{G}=-2$ (*1/4×*), etc.

All metrics were averaged over the 200 replicas per $(\tau,\mu)$; detailed results appear in the Supplementary Information.

#### Coexistence as the main attractor

A central objective of our multispecies model is to explore how the introduction of an engineered plasmid influences the structure and dynamics of a complex microbial community. This question is closely tied to a long-standing concern in ecological engineering: the risk of negative cascading effects when introducing modified organisms into the wild. As highlighted in [28, 49], one proposed strategy to mitigate such risks is to minimize ecological disruption by engineering strains that are derived from wild-type species already present in the community. In the absence of horizontal gene transfer (HGT), this approach appears relatively safe, since the modified strain remains confined to a specific ecological role shared by its wild-type counterpart. However, the introduction of HGT as a tool for ecosystem engineering could change the landscape. With HGT, genetically modified material can spread across multiple species boundaries, potentially reshaping ecological networks in ways that are difficult to anticipate. As shown below, the mathematical model strongly points otherwise to a robust community response with no biodiversity loss and improved functionality.

**Fig. 4:**
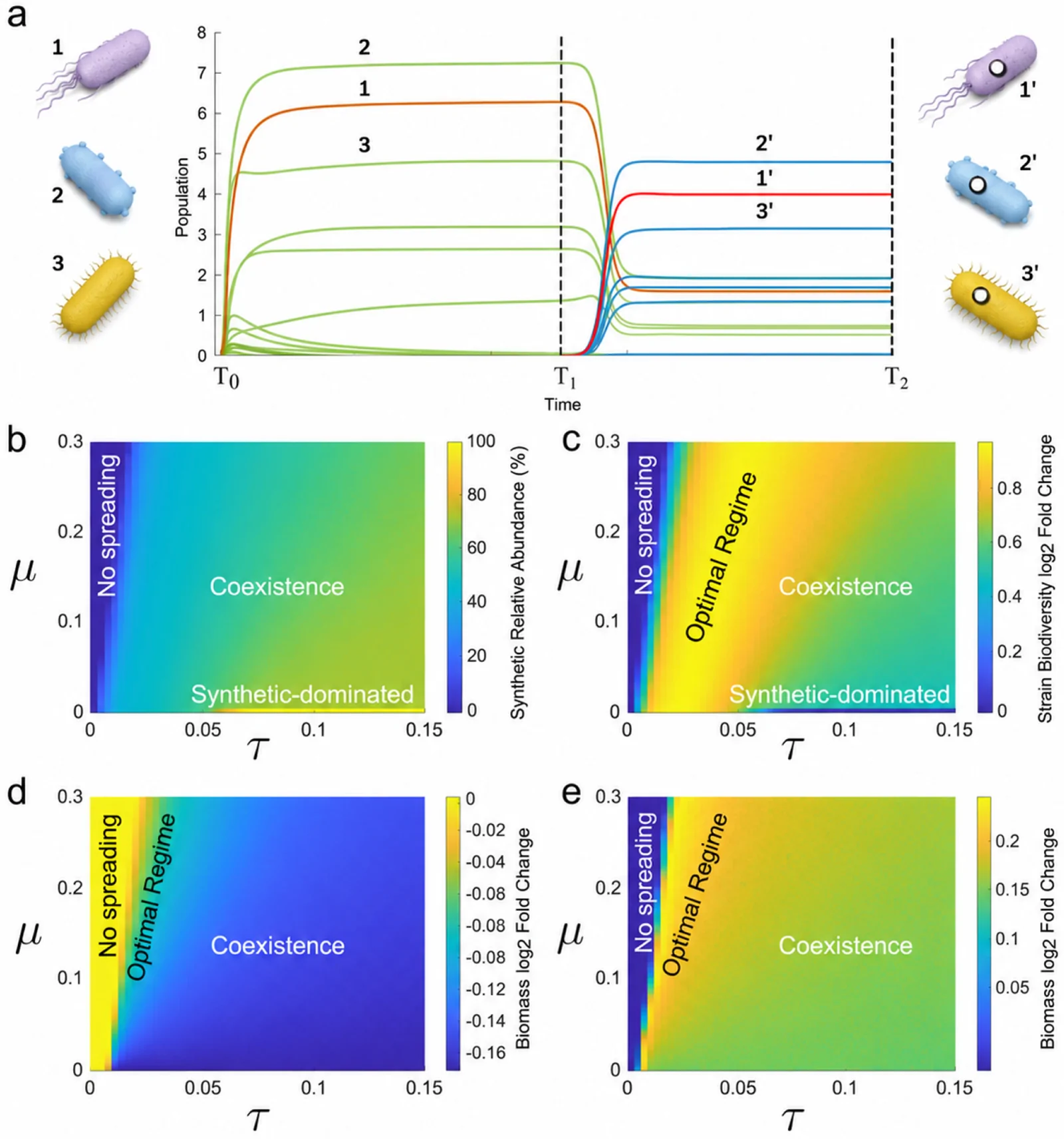
Statistical analysis of the high-dimensional model for $\eta^{\prime} = \eta - 0.1\eta$ for a community with *n=m=20*. After the first inoculation of the synthetic plasmid carrier at $t=T_1$, a new stability state is reached at $t=T_2$. Panel (**a**) illustrates the assembly process to $T_1$, with color coding for wild-type (green), synthetic (blue), initial wild-type chosen plasmid carrier (orange), and its synthetic counterpart (red). In (**b**), the relative abundance of $S_i$ vs $N_i$ and in (**c**) The biodiversity Fold change across *τ* and *μ* values are shown, both results at $t=T_2$. Panel (**d**) mirrors (**c**) but with biomass Fold change. Panel (**e**) also shows biomass Fold change at $t=T_2$, but in this case, the totality of the resources is positively affected by the synthetic species, likewise following Eq. 4. Here above-zero Fold change values are observed despite metabolic burden drawbacks. Interestingly, in all cases (b,c, and d), a sweet spot in terms of biodiversity and biomass production appears near the transition point. The results remain consistent across parameters, here with randomized values: $\rho_k \in [0.8, 1]$, $\beta_k \in [0.7, 0.9]$, *γ = 0.3*, $\eta_i \in [0.5, 1]$, $\eta^{\prime} = \eta - 0.1\eta$ and $\sigma_i \in [0.5, 0.7]$

One way to quantify structural changes in the community after HGT is by measuring the Kullback–Leibler (KL) divergence, which captures how much one distribution differs from another, in this case, the relative abundances of strains before and after inoculation. Intuitively, a low KL value means that the overall community composition remains similar. Across simulations, KL values remain consistently low (see SM), suggesting that, although individual biomasses and strain identities may change (see the sections below), the broader structure of the community and the coexistence attractor remain stable. For instance, if the original community was dominated by strains 2, 1, and 3, the new configuration typically retains a similar profile, now possibly dominated by the plasmid-carrying variants 2’, 1’, and 3’ (Fig. 4a).

Once again, the coexistence attractor, where both wild-type and synthetic strains are present, dominates the space of possible outcomes (Fig. 4b). Wild-type strains only fully disappear in the extreme case where *μ=0* and the plasmid is never lost. Interestingly, in the multispecies context, plasmid spread increases the space of coexistence compared to the low-dimensional model. Because HGT acts as an epidemic process between species, having more potential host species increases the effective transmission rate (see SM). This, in turn, makes the spread of the plasmid easier: Even a small transmission rate *τ* - both within and between species - quickly ensures the success of the HGT intervention and allows the long-term coexistence of synthetic and wild-type strains.

#### Strain biodiversity increase

At *τ = 0*, the plasmid cannot propagate, and only wild-type strains persist. In contrast, when *μ = 0*, the plasmid is never lost and synthetic variants fully occupy the system. Between these extremes, we observe varying degrees of coexistence. Here, we ask whether this coexistence increases the overall biodiversity of the strain, that is, the total number of distinct strains, or whether some species are lost in the process.

A central result of our analysis is that, if $m^*$ denotes the number of wild-type species that survive in steady state before inoculation of the plasmid, then the total biodiversity of the system is always equal to or greater than $m^*$. In fact, biodiversity can reach $2m^*$, resulting in a two-fold increase in strain diversity in the limit-case scenario where all wild-types and synthetic strains coexist (Fig. 4c; see Methods and SM). At other the two limiting cases (*τ = 0* or *μ = 0*), the community contains either $m^*$ wild-type strains or $m^*$ synthetic variants, respectively. However, in the intermediate regime, we consistently find more than $m^*$ strains, indicating that some species persist in both wild-type and synthetic forms.

At high transmissibility (*τ*), all synthetic strains are present, along with a subset of the most abundant wild types. In this case, rarer wild-type strains are typically replaced by their plasmid-carrying counterparts (see SM for a detailed analysis). In contrast, at intermediate values of *τ*, the system reaches maximum strain diversity, with all species maintaining both wild-type and synthetic forms. As predicted by the low-dimensional model, this result shows that a high *τ* is not required for a successful HGT intervention. Instead, intermediate values of *τ* define an optimal regime, where synthetic strains are effectively introduced without displacing their wild-type counterparts.

#### Total biomass gain

Synthetic variants carry an inherent trade-off: While they can reduce resource degradation (parameterized by *γ*), one can expect that this might induce them to be less efficient consumers, with $\eta^{\prime} < \eta$. This raises a key ecological question: Does the introduction of synthetic strains lead to a net increase in total community biomass due to improved resource conservation, or does it reduce biomass as a result of diminished replication efficiency? To investigate this, we quantify the fold change in total biomass from time $T_1$ to time $T_2$, as described in the Materials and Methods section. This metric captures the net impact of engineering interventions on community productivity.

Our analysis reveals a clear pattern. When the engineered plasmid targets a single resource, the biomass fold change is generally negative across most of the coexistence region (Fig. 4d), indicating a net loss in total biomass. This suggests that, under this minimal engineering scenario, the cost of carrying the plasmid outweighs its ecological benefit. This outcome aligns with a conservative baseline assumption, where the metabolic burden imposed by reduced efficiency dominates over the limited savings in resources. However, there is a notable exception: Near the optimal parameter regime discussed earlier, the biomass loss is less pronounced. This is visible in Fig. 4d as light green areas, where total biomass remains close to or slightly above baseline levels. In these cases, most individuals in the community remain wild-type, and only a small fraction carries the synthetic plasmid, minimizing the collective cost.

But what if the engineered function affects multiple resources simultaneously? Consider, for example, encoding a gene that synthesizes a hygroscopic molecule such as hyaluronic acid, which enhances water retention in the environment. This could not only improve access to water, but also indirectly improve the availability of several other resources [46, 47, 50, 51]. This multiresource enhancement scenario is explored in detail in the SM, where we systematically vary the number of resources affected by the engineered function, from one to all.

As shown in Fig. 4e, when all resources are positively impacted by the engineered strains, the change in biomass fold is consistently positive across the entire co-existence region. This indicates a robust ecological benefit of the intervention: even at low values of plasmid transmission rate *τ*, the engineered function promotes higher total biomass without compromising species coexistence. This result underscores the potential of synthetic strategies that enhance broad ecological functions rather than narrowly targeting individual resources.

Importantly, the previously identified optimal region reemerges clearly under this expanded scenario, reaffirming its role as a balance point between metabolic cost and community-wide benefit. In addition, the strain diversity remains stable regardless of the number of resources targeted, as it is primarily governed by the dynamics of horizontal plasmid transfer, a result consistent with both the low-dimensional model and the full simulations discussed above.

#### Optimal regime

The presence of an optimal regime is not an obvious feature and warrants careful consideration. The key to understanding it is twofold. From the perspective of the pure dynamics of plasmid spreading, there is a peak in strain biodiversity when the ratio $\tau/\mu$ is low. This scenario maintains a proportionally small number of synthetic individuals in the community; therefore, the metabolic burden, given by $\eta^{\prime} = \eta - 0.1\eta$, has a reduced impact on the general community, and low-abundance species, which are more susceptible, are less stressed.

Secondly, when the number of affected resources is sufficiently high, biomass gain is optimized by a synthetic community that is small enough to avoid triggering a global loss due to metabolic burden, yet large enough to produce a meaningful improvement in resource dampening. This trade-off is clearly evident in Eq. 4. Resources are depleted through both consumption and natural decay, as described in Eq. 1. Importantly, the decay term in Eq. 4 is modulated by the presence of the synthetic community, introducing a dampening dynamic. If we evaluate the following limit:

22 $$\lim_{\sum_{i=1}^{n} S_i \to \infty}F(R_k,\mathbf{S})=\lim_{\sum_{i=1}^{n} S_i \to \infty}\frac{\beta_k R_k}{1+\delta_k\displaystyle\frac{\sum_{i=1}^{n}S_i}{\gamma}}$$

Observe that as $\sum_{i=1}^{n} S_i \to \infty$, that is, as the synthetic community reaches its maximum size, the denominator behaves as

23 $$1+\delta_k \displaystyle\frac{\sum_{i=1}^{n}S_i}{\gamma} \rightarrow \begin{cases} \infty & \text{if } \delta_k = 1,\\ 1 & \text{if } \delta_k = 0.\end{cases}$$

Therefore, the limit becomes

24 $$\lim_{\sum_{i=1}^{n} S_i \to \infty} F(R_k,\mathbf{S}) = \begin{cases} 0 & \text{if } \delta_k = 1,\\ \beta_k R_k & \text{if } \delta_k = 0.\end{cases}$$

When a resource is affected, its depletion cannot fall below its consumption rate, leading to a plateau in the potential gain. Expanding the synthetic community beyond a certain point does not proportionally increase the positive effect on resource availability. Instead, an optimal balance, or optimal regime, can be achieved by tuning, through design, the plasmid transmissibility and/or plasmid loss rates [29]. This conclusion is robust to the choice of a reasonable resource-modification function: a capped linear, less restrictive formulation of F yields the same qualitative effect (SM).

#### Robustness to ecological and conjugation heterogeneity

**Fig. 5:**
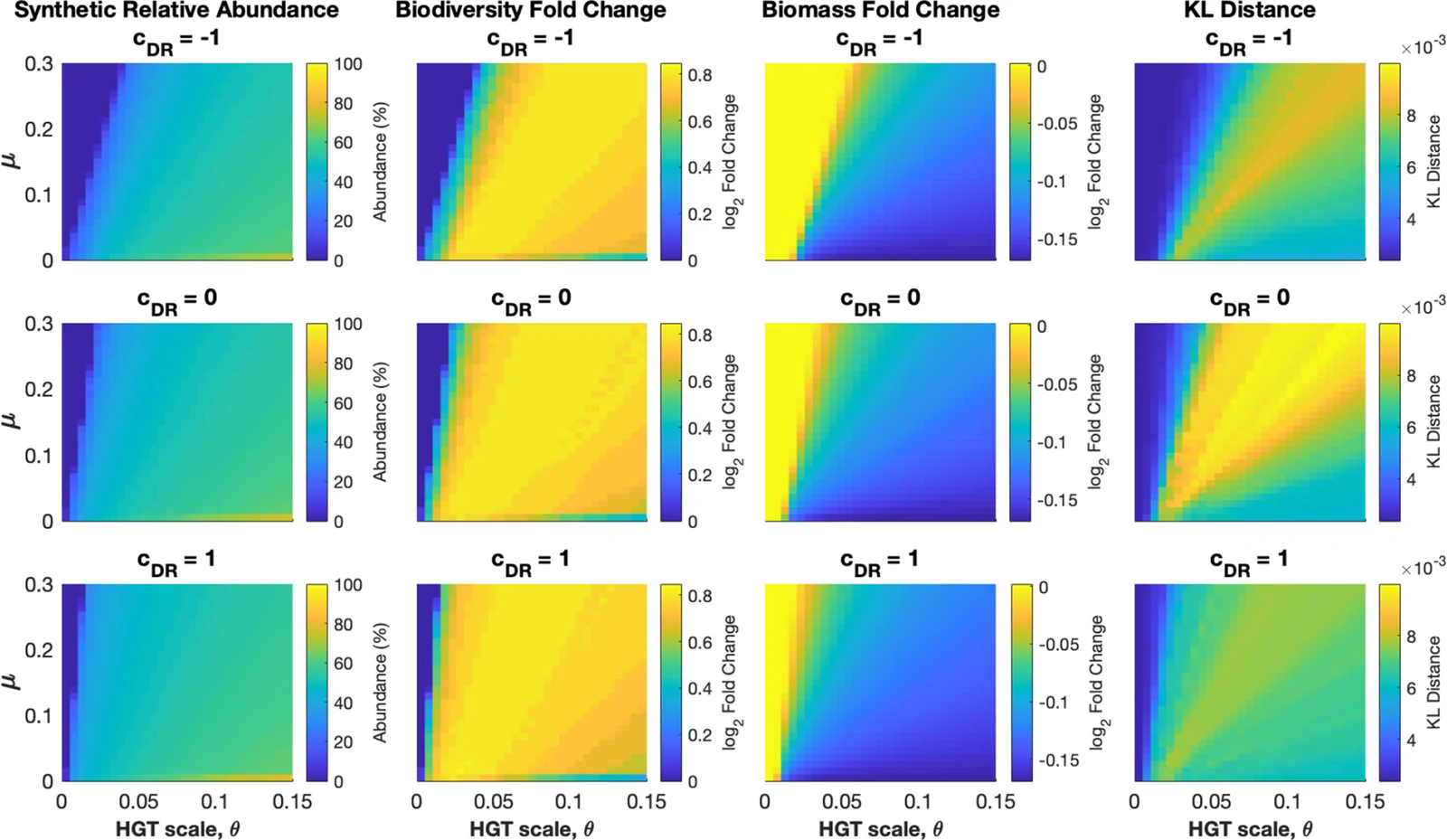
Robustness of HGT-mediated ecosystem engineering under heterogeneous donor and recipient abilities. Donor ($d_i$) and recipient ($r_i$) abilities were drawn from correlated lognormal distributions, with donor-recipient correlation coefficients $c_{DR}=-1$, *0*, and *1* (rows). For each parameter combination, the median across independent realizations is shown. Columns report the relative abundance of plasmid-bearing cells, biodiversity fold change, biomass fold change, and the Kullback-Leibler distance between the pre- and post-intervention communities as functions of the HGT scale (*θ*) and plasmid loss rate (*μ*). Heterogeneous transfer networks modify the quantitative details of the ecological response, particularly the extent of community restructuring, while preserving the principal qualitative outcomes. Coexistence remains widespread, biodiversity increases, biomass responses remain contingent on the number of targeted resources, and community restructuring remains limited, especially when $c_{DR}=1$

The qualitative behavior of the model remains remarkably robust after introducing ecological and conjugation heterogeneity. The three main results, namely coexistence between plasmid-bearing and plasmid-free strains, the increase in strain-level diversity and the emergence of an optimal transmission regime, all persist across the explored scenarios (Fig. 5).

Allowing plasmid-bearing strains to differ from their wild-type counterparts through distinct resource-consumption profiles primarily affects the magnitude of community reorganization. Increasing ecological heterogeneity progressively alters the Kullback-Leibler distance between the initial and final community compositions, indicating that stronger ecological differentiation can lead to greater shifts in taxonomic composition and relative abundances. This is an expected result of the limit-case scenario where the introduced synthetic species are no longer similar to wild-type counterparts, but rather novel invasive species. Nevertheless, the central outcomes of the model remain unchanged. Across all heterogeneity levels, horizontal gene transfer continues to promote coexistence between plasmid bearers and wild types and keeps increasing strain-level diversity within an optimal range (SM).

Introducing species-specific donor and recipient abilities produces similarly robust results. Communities characterized by positive donor-recipient correlations ($c_{DR}=1$), corresponding to the presence of *superspreaders*, consistently exhibit the lowest Kullback-Leibler distances. This indicates that efficient plasmid dissemination can be achieved with comparatively smaller departures from the original community composition. By contrast, communities with independent ($c_{DR}=0$) or negatively correlated ($c_{DR}=-1$) donor and recipient traits exhibit larger compositional shifts despite displaying similar coexistence and diversity maintenance patterns (Fig. 5). This highlights the importance of identifying species with both high $r_i$ and $d_i$ parameters for efficient spreading.

Because textitsuperspreader species dominate horizontal gene transfer, efficient plasmid dissemination can be achieved without requiring widespread plasmid carriage throughout the entire community. As a result, the metabolic burden associated with plasmid maintenance is concentrated in a subset of species rather than being distributed across all community members. This creates a favorable balance between plasmid transmission and plasmid-associated costs, facilitating plasmid spread while minimizing ecosystem-wide reorganization.

This observation resonates with the ecological concept of ecosystem engineers [52], where a small number of species exert disproportionately large effects on ecosystem structure and function. In our model, superspreaders act as key transmission hubs that mediate plasmid dissemination while minimizing broader community disruption. From an applied perspective, this suggests that targeting highly connected species may provide an efficient strategy for introducing engineered functions into complex microbial communities while limiting unintended ecological perturbations.

## Discussion

Could horizontal gene transfer be harnessed as a safe and effective tool for ecosystem restoration? Our results suggest that this strategy is theoretically feasible: When applied with precision, synthetic biology could enhance and stabilize microbial communities through horizontal gene transfer (HGT) while largely preserving ecological balance. Inspired by soil microbiomes as a general case study, we demonstrate that engineered strains carrying functional plasmids can be successfully spread through microbial networks under controlled conditions. This strategy not only preserves strain-level biodiversity but can, in some cases, also lead to a net increase in total community biomass.

A simplified analytical model recovers the classical result that the balance between plasmid transmissibility (*τ*) and loss rate (*μ*) governs whether plasmids can successfully establish and persist within a community [37]. When $\tau/\mu$ exceeds a critical threshold, coexistence between synthetic and wild-type strains becomes the dominant outcome, a result that aligns well with the behavior observed in more detailed, multidimensional simulations. Building upon this foundation, our multidimensional consumer-resource model shows how these established HGT dynamics translate into ecosystem-level responses when plasmids encode engineered ecological functions.

Importantly, using native species as initial plasmid carriers ensures that synthetic strains remain ecologically compatible, occupying the same resource utilization profile as their wild-type counterparts. This design principle prevents major changes in community composition, as confirmed by negligible Kullback–Leibler divergence before and after synthetic introduction. Coexistence emerges as the primary attractor in a wide range of conditions, with plasmid spread limited by loss rates and competition arising from the shared resource-consumption profiles of plasmid-bearing and plasmid-free strains, thereby avoiding disruptive dominance by engineered variants. In practice, this suggests not maximal spread, but rather constructs whose conjugation range and frequency are sufficient to offset realistic plasmid loss. These conclusions remain robust under moderate perturbations of the resource uptake profiles of engineered strains and under heterogeneous donor and recipient transfer abilities. Heterogeneous transfer networks further reveal that a small number of highly effective donor species can act as key disseminators of engineered functions, suggesting that HGT network structure itself may represent a design variable for ecosystem engineering. As engineered strains progressively diverge from the resource uptake profiles of their wild-type counterparts, however, community restructuring becomes increasingly pronounced, approaching the dynamics expected from introducing ecologically distinct species.

HGT systematically increases strain-level biodiversity (Fig. 4c). Each successful transfer adds a synthetic counterpart, usually without displacing its original wild-type ancestors. The central idea is that HGT does not simply add new competing species to the system, but rather synthetic variants that share the same resource profiling as their wild-type, while increasing resource retention. This strain diversification peaks within an “optimal region” in parameter space, where propagation is efficient but controlled, and the metabolic cost of plasmid carriage remains modest. As a result, a modest but persistent plasmid presence is sufficient to increase resource availability and biodiversity while avoiding unnecessary metabolic costs.

The targeting of functions is key to success. When the engineered cassette buffers only a single limiting resource, biomass responses are modest or even slightly negative, as the metabolic burden of carrying the new plasmid offsets the ecological benefit. In contrast, when the cassette acts as a *keystone gene*, that is, one whose expression simultaneously affects multiple limiting factors, the effects become pronounced at the community level. Hygroscopic exopolymers provide a concrete example: By retaining water, they indirectly stabilize several resources at once, shifting the system toward higher biomass across a broad coexistence region (Fig. 4e). More generally, engineered functions that couple to systemic constraints, such as hydrology, nutrient retention, or pH buffering, are likely to outperform narrowly targeted constructs, as they relieve integrated ecological bottlenecks rather than isolated ones.

These properties also have safety implications. First, because the coexistence phase is a generic outcome of the model, wild-type strains tend to persist unless plasmid loss becomes negligible ($\mu \to 0$). As a result, complete replacement of wild types is unlikely under realistic conditions. Second, the saturating form of the function *F* imposes diminishing returns: increasing transmissibility beyond the optimal range provides little additional benefit while increasing the metabolic burden. Furthermore, the functional form of *F* captures the decay-damping effect on a targeted resource, which represents one of the most stringent scenarios. Other functional mechanisms, such as detoxification, post-fire alkalinity buffering, or heavy metal remediation, may couple differently to resource dynamics and should be examined in future studies.

In summary, by coupling consumer-resource dynamics with the epidemic-like spreading of synthetic genes, our framework provides a theoretical basis for exploring HGT as a mechanism for ecosystem engineering. The model identifies conditions under which engineered ecological functions can disseminate through native microbial communities, triggering beneficial ecosystem responses while largely preserving their ecological structure. Our results identify qualitative conditions under which engineered ecosystem functions can successfully disseminate while minimizing ecological disruption. Future work should explore spatial heterogeneity, evolutionary feedbacks, additional ecological interactions such as cross-feeding and inhibition, and experimental validation to assess long-term viability. Overall, our findings offer a firt step toward a design roadmap for the deployment of engineered microbes in support of ecosystem resilience and restoration.

## Electronic supplementary material

Below is the link to the electronic supplementary material.

Supplementary Material 1

Supplementary Material 2

## Data Availability

No datasets were generated or analysed during the current study.
