# Supplementary material for: Synthetic horizontal gene transfer for ecosystem restoration
Source: Biol Direct. 2026 Aug 14;21:201. doi: 10.1186/s13062-026-00919-9 (PMC13662984; doi:10.1186/s13062-026-00919-9)
Supplement: Supplementary file 2 [file 13062_2026_919_MOESM2_ESM.pdf]

Synthetic Horizontal Gene Transfer for Ecosystem Restoration

Supplementary Material

Victor Maull *,[1,2] Guim Aguadé-Gorgorió,[3] Victor de Lorenzo,[4] and Ricard Solé †[1,2,5]

[1] *ICREA-Complex Systems Lab, University Pompeu Fabra, Dr Aiguader 80, 08003 Barcelona*
[2] *Institut de Biologia Evolutiva, CSIC-UPF, Pg Maritim de la Barceloneta 37, 08003 Barcelona*
[3] *ISEM, University Montpellier, CNRS, IRD, Montpellier, France*
[4] *Centro Nacional de Biotecnologia, CSIC, Madrid, Spain*
[5] *Santa Fe Institute, 1399 Hyde Park Road, Santa Fe NM 87501, USA*

Keywords: Ecological networks, bioengineering, synthetic biology, invasion dynamics

Contents

Stability analysis of the three–dimensional mean–field model 1

Plasmid transfer in a simple two-species model: Epidemiologic Approach 4
Single species HGT 4
Two species HGT, no promiscuity 5
Two species HGT and promiscuity 6
The difference with classical SIS models 7

High-Dimensional Model Analysis 8
Community Structure 8
Varying the Number of Targeted Resources 10
Fully Randomized Parameter Regime 12

Stability analysis of the three–dimensional mean–field model

We consider the three–dimensional mean–field system

$$\frac{dR}{dt} = m\bar{\rho} - RN - RS - \frac{\bar{\beta}R}{1+S/\gamma}, \tag{1}$$

$$\frac{dN}{dt} = \bar{\eta}NR - \tau NS + \mu S - \sigma N, \tag{2}$$

$$\frac{dS}{dt} = \bar{\eta}' SR + \tau NS - \mu S - \sigma S. \tag{3}$$

The variables R, N, and S denote the resource, the wild–type population, and the synthetic strain, respectively. All parameters are taken as nonnegative constants.

Derivation of the fixed points. Fixed points satisfy $dR/dt = dN/dt = dS/dt = 0$, that is,

$$0 = m\bar{\rho} - R^*N^* - R^*S^* - \frac{\bar{\beta}R^*}{1+S^*/\gamma}, \tag{4}$$

$$0 = \bar{\eta}N^*R^* - \tau N^*S^* + \mu S^* - \sigma N^*, \tag{5}$$

$$0 = \bar{\eta}' S^*R^* + \tau N^*S^* - \mu S^* - \sigma S^*. \tag{6}$$

* Corresponding author
† Corresponding author

We first look for a purely resource fixed point. Setting $N^* = 0$ and $S^* = 0$ in (4) gives

$$0 = m\bar{\rho} - \frac{\bar{\beta} R_0^*}{1 + 0/\gamma} = m\bar{\rho} - \bar{\beta} R_0^*. \tag{7}$$

Therefore

$$R_0^* = \frac{m\bar{\rho}}{\bar{\beta}}, \tag{8}$$

and we obtain

$$P_0 = (R_0^*, 0, 0). \tag{9}$$

Next we seek a fixed point with wild type present and no synthetic strain, i.e. $S^* = 0$ and $N^* > 0$. Equation (6) is then trivially satisfied. From (5) with $S^* = 0$ we obtain

$$0 = \bar{\eta} N_1^* R_1^* - \sigma N_1^* = N_1^* \left(\bar{\eta} R_1^* - \sigma \right). \tag{10}$$

Since we assume $N_1^* > 0$, we must have

$$\bar{\eta} R_1^* - \sigma = 0, \tag{11}$$

and hence

$$R_1^* = \frac{\sigma}{\bar{\eta}}. \tag{12}$$

Substituting this into (4) with $S^* = 0$ gives

$$0 = m\bar{\rho} - R_1^* N_1^* - \bar{\beta} R_1^*, \tag{13}$$

or

$$R_1^* N_1^* = m\bar{\rho} - \bar{\beta} R_1^*. \tag{14}$$

Using $R_1^* = \sigma/\bar{\eta}$ we obtain

$$N_1^* = \frac{m\bar{\rho} - \bar{\beta} R_1^*}{R_1^*} = \frac{m\bar{\rho}\bar{\eta}}{\sigma} - \bar{\beta}. \tag{15}$$

Thus

$$P_1 = \left(R_1^*, N_1^*, 0 \right), \quad R_1^* = \frac{\sigma}{\bar{\eta}}, \quad N_1^* = \frac{m\bar{\rho}\bar{\eta}}{\sigma} - \bar{\beta}. \tag{16}$$

Biological feasibility requires $N_1^* > 0$, which constrains the parameters.

Finally, we consider the coexistence fixed point P_c, for which $N_c^* > 0$ and $S_c^* > 0$. In this case, both (5) and (6) contain a factor N^* or S^*, and we can divide by them. From (5) we write

$$0 = \bar{\eta} N_c^* R_c^* - \tau N_c^* S_c^* + \mu S_c^* - \sigma N_c^*, \tag{17}$$

and divide by $N_c^* > 0$:

$$0 = \bar{\eta} R_c^* - \tau S_c^* + \mu \frac{S_c^*}{N_c^*} - \sigma. \tag{18}$$

From (6) we obtain

$$0 = \bar{\eta}' S_c^* R_c^* + \tau N_c^* S_c^* - \mu S_c^* - \sigma S_c^*, \tag{19}$$

and dividing by $S_c^* > 0$ gives

$$0 = \bar{\eta}' R_c^* + \tau N_c^* - \mu - \sigma. \tag{20}$$

Equation (20) can be solved for R_c^* as

$$R_c^* = \frac{\mu + \sigma - \tau N_c^*}{\bar{\eta}'}. \tag{21}$$

Substituting (21) into (18) provides a relation between N_c^* and S_c^*. At the same time, (4) with $N_c^* > 0$, $S_c^* > 0$ fixes R_c^* through

$$m\bar{\rho} = R_c^* N_c^* + R_c^* S_c^* + \frac{\bar{\beta} R_c^*}{1 + S_c^*/\gamma}. \tag{22}$$

Equations (21)–(22) define the coexistence fixed point $P_c = (R_c^*, N_c^*, S_c^*)$. Closed expressions are lengthy and not required for the stability boundary; it is enough to note that, whenever a positive solution exists with $R_c^* > 0$, $N_c^* > 0$, $S_c^* > 0$, the three populations coexist.

Absence of a purely synthetic equilibrium. We now show that a fixed point with $N^* = 0$ and $S^* > 0$ does not exist for $\mu > 0$. Assume $N^* = 0$, $S^* > 0$. Equation (5) becomes

$$0 = \mu S^*, \tag{23}$$

which implies either $\mu = 0$ or $S^* = 0$. For $\mu > 0$, this is only compatible with $S^* = 0$, contradicting our assumption $S^* > 0$. Therefore, for $\mu > 0$, no equilibrium with $N^* = 0$, $S^* > 0$ exists. In mean field, the synthetic strain cannot fully displace the wild type. Plasmid loss thus stabilizes coexistence, and only in the singular limit $\mu = 0$ can a full replacement of the wild type occur.

Jacobian matrix The Jacobian matrix of the vector field $(\dot{R}, \dot{N}, \dot{S})$ is

$$J(R, N, S) = \begin{pmatrix} J_{11} & -R & J_{13} \\ \bar{\eta} N & \bar{\eta} R - \tau S - \sigma & -\tau N + \mu \\ \bar{\eta}' S & \tau S & \bar{\eta}' R + \tau N - \mu - \sigma \end{pmatrix}, \tag{24}$$

with

$$J_{11} = -N - S - \frac{\bar{\beta}}{1 + S/\gamma} + \frac{\bar{\beta} R S}{\gamma (1 + S/\gamma)^2}, \tag{25}$$

$$J_{13} = -R + \frac{\bar{\beta} R}{\gamma (1 + S/\gamma)^2}. \tag{26}$$

For a given fixed point $P^* = (R^*, N^*, S^*)$, we introduce small deviations $\delta \mathbf{x} = (\delta R, \delta N, \delta S)^\top$. To linear order,

$$\frac{d}{dt} \delta \mathbf{x} = J(P^*)\, \delta \mathbf{x}. \tag{27}$$

Let λ_i and $\mathbf{v}_i$ denote the eigenvalues and eigenvectors of $J(P^*)$, with $i = 1, 2, 3$.

Stability of the resource–only fixed point. At $P_0 = (R_0^*, 0, 0)$, we have $R_0^* = m\bar{\rho}/\bar{\beta}$, $N^* = 0$, $S^* = 0$. The Jacobian simplifies to

$$J(P_0) = \begin{pmatrix} -\bar{\beta} & -R_0^* & -R_0^* \\ 0 & \bar{\eta} R_0^* - \sigma & \mu \\ 0 & 0 & \bar{\eta}' R_0^* - \mu - \sigma \end{pmatrix}. \tag{28}$$

The eigenvalues are immediately

$$\lambda_1 = -\bar{\beta}, \quad \lambda_2 = \bar{\eta} R_0^* - \sigma, \quad \lambda_3 = \bar{\eta}' R_0^* - \mu - \sigma. \tag{29}$$

Thus P_0 is stable only if $\bar{\eta} R_0^* < \sigma$ and $\bar{\eta}' R_0^* < \mu + \sigma$, i.e. neither population can grow on the supplied resource. Whenever $\bar{\eta} R_0^* > \sigma$ or $\bar{\eta}' R_0^* > \mu + \sigma$, at least one eigenvalue is positive and P_0 is unstable.

Stability of the wild–type fixed point and critical line. At $P_1 = (R_1^*, N_1^*, 0)$, with $R_1^* = \sigma/\bar{\eta}$, $N_1^* = m\bar{\rho}\bar{\eta}/\sigma - \bar{\beta}$, we obtain

$$J(P_1) = \begin{pmatrix} -N_1^* - \bar{\beta} & -R_1^* & -R_1^* \\ \bar{\eta} N_1^* & \bar{\eta} R_1^* - \sigma & -\tau N_1^* + \mu \\ 0 & 0 & \bar{\eta}' R_1^* + \tau N_1^* - \mu - \sigma \end{pmatrix}. \tag{30}$$

The matrix is block triangular, and the eigenvalue controlling the invasion of the synthetic strain is the lower–right entry:

$$\lambda_S = \bar{\eta}' R_1^* + \tau N_1^* - \mu - \sigma. \tag{31}$$

Substituting $R_1^* = \sigma/\bar{\eta}$, we write

$$\lambda_S = \left(\frac{\bar{\eta}'}{\bar{\eta}} - 1\right)\sigma + \tau N_1^* - \mu. \tag{32}$$

The wild–type fixed point P_1 is stable against invasion of S if and only if $\lambda_S < 0$, and unstable if $\lambda_S > 0$.

The loss of stability of P_1 marks a transcritical bifurcation where P_1 and P_c exchange stability. The critical line is obtained by imposing $\lambda_S = 0$, which yields

$$\mu_c(\tau) = \left(\frac{\bar{\eta}'}{\bar{\eta}} - 1\right)\sigma + N_1^* \tau. \tag{33}$$

For $\mu < \mu_c(\tau)$, the coexistence state P_c is stable and the synthetic strain persists. For $\mu > \mu_c(\tau)$, P_1 is stable and the synthetic strain cannot be maintained.

Plasmid transfer in a simple two-species model: Epidemiologic Approach

One mechanism by which bacteria can share genetic material is through horizontal gene transfer (HGT). We have a system in which a plasmid is engineered so that it can be transferred between bacteria of different species. Before implementing the complete model in which different species compete for resources while undergoing plasmid transfers, we can study the model with transfers alone. The fundamental question here is: how will *promiscuity*, the capacity of the plasmid to jump between different species, alter the outcomes of the HGT process? To answer this question, we will make simplifications to the original problem, for example, reducing the complexity of the consumer-resource model into a simple model in which species grow up to a certain carrying capacity without additional interactions.

The phenomenon under study is similar to a Susceptible-Infected-Susceptible epidemic model, yet with particularities in how infection rates are modeled. In this document "infection" refers to receiving the plasmid, and "recovery" refers to losing it and becoming wild-type again. The particularity of the exercise here is that, to make two species "different" and analyse how they are each affected by HGT from the other species, we can no longer use the model of relative abundances summing to 1, $N_i + S_i = 1$, but we have to write the model in terms of different carrying capacities, $N_i + S_i = K_i$.

Beyond the numerical and some analytical results, I also provide some discussion comparing the model we use to epidemic models where infections are frequency- and not abundance-dependent.

Single species HGT

We label the wild-type species that does not carry the engineered plasmid N_i, and the equivalent species with the plasmid is S_i. Here we decide that the total populations, defined by capital letters, fulfill $N_1 + S_1 = K_1 \neq 1$, so that we introduce a carrying capacity K_i as a measure of how *fit* species 1 is. In the context of the full model involving resource competition, K_1 is a property of how species 1 consumes the resources. One could write, for example

$$\frac{dN_1}{dt} = r_1 N_1 \left(1 - \frac{N_1 + S_1}{K_1}\right) - \tau N_1 S_1 + \mu S_1, \tag{34}$$

$$\frac{dS_1}{dt} = r_1' S_1 \left(1 - \frac{N_1 + S_1}{K_1}\right) + \tau N_1 S_1 - \mu S_1. \tag{35}$$

Here, r_1 and r_1' are the replication rates of the species, which could be lower for the individuals carrying the plasmid, K_1 is the carrying capacity, τ is the rate of HGT (infection) after an $N_1 S_1$ encounter, and μ is the rate at which the plasmid is lost (recovery). It is easy to see that the first term related to replication will vanish at equilibrium, because

$$\frac{dN_1}{dt} + \frac{dS_1}{dt} = (r_1 N_1 + r_1' S_1)\left(1 - \frac{N_1 + S_1}{K_1}\right), \tag{36}$$

from where

$$N_1^* + S_1^* = K_1. \tag{37}$$

Because of this, to study the distribution of wild-type and infected populations at equilibrium, it is sufficient to study

$$\frac{dN_1}{dt} = -\tau N_1 S_1 + \mu S_1, \tag{38}$$
$$\frac{dS_1}{dt} = +\tau N_1 S_1 - \mu S_1. \tag{39}$$

This is very similar to the classical SIS model, yet here infections are not proportional to the fraction of infected individuals, but to the total population. See below for a discussion about this difference.

In any case, by introducing $S_1 = K_1 - N_1$ we can write

$$\frac{dN_1}{dt} = -\tau N_1(K_1 - N_1) + \mu(K_1 - N_1) \tag{40}$$

The solution of the model is $N_1^* = \frac{\mu}{\tau}$ and $S_1^* = K_1 - \frac{\mu}{\tau}$. Because infections are proportional to the total number of infected individuals, a larger carrying capacity K_1 results in a larger infected population, whereas the wild type population is not proportional to the carrying capacity, but only to the recovery-to-infection rate.

This somehow strange result, arising from the difference with the SIS model discussed below, is what had me doubting. In the SIS model, both populations are a fraction of the total population (see final section). I am now convinced that this is the right way to study the problem (and is consistent with the model you work with).

Two species HGT, no promiscuity

Now, assume that we have two species in place, each with its own HGT process. Because we want to focus on a minimal model capturing the HGT process, let's assume that species do not compete for resources or space for now, and so there are no interactions whatsoever. This is, of course, a preliminary simplification of the final model in which species not only undergo HGT but also compete for a pool of available resources, constraining the options for multispecies coexistence.

In the absence of interactions nor promiscuous (inter-species) HGT, the model simply writes

$$\frac{dN_1}{dt} = -\tau N_1 S_1 + \mu S_1, \tag{41}$$
$$\frac{dS_1}{dt} = +\tau N_1 S_1 - \mu S_1, \tag{42}$$
$$\frac{dN_2}{dt} = -\tau N_2 S_2 + \mu S_2, \tag{43}$$
$$\frac{dS_2}{dt} = +\tau N_2 S_2 - \mu S_2 \tag{44}$$

And with the same procedure as above, we simplify to

$$\frac{dN_1}{dt} = -\tau_1 N_1(K_1 - N_1) + \mu_1(K_1 - N_1), \tag{45}$$
$$\frac{dN_2}{dt} = -\tau_2 N_2(K_2 - N_2) + \mu_2(K_2 - N_2) \tag{46}$$

from where it is obvious to find

$$\left(N_1^* = \frac{\mu_1}{\tau_1}, S_1^* = K_1 - \frac{\mu_1}{\tau_1}\right), \tag{47}$$
$$\left(N_2^* = \frac{\mu_2}{\tau_2}, S_2^* = K_2 - \frac{\mu_2}{\tau_2}\right). \tag{48}$$

Each species will have a fraction of individuals carrying the plasmid proportional to its carrying capacity and the recovery-to-infection rate. A species with a higher total population, or a higher HGT/infection rate, will have a higher population of individuals carrying the plasmid. This simply results from the higher number of infectious, plasmid-carrying individuals present.

Two species HGT and promiscuity

The central part of our study comes when one considers the possibility that engineered plasmids can jump between species. Now, each species can become infected (receive the plasmid) after contact with an individual from each of the two species. Mathematically, this can be simply written as

$$\frac{dN_1}{dt} = -\tau N_1(S_1 + S_2) + \mu S_1, \tag{49}$$
$$\frac{dS_1}{dt} = +\tau N_1(S_1 + S_2) - \mu S_1, \tag{50}$$
$$\frac{dN_2}{dt} = -\tau N_2(S_1 + S_2) + \mu S_2, \tag{51}$$
$$\frac{dS_2}{dt} = +\tau N_2(S_1 + S_2) - \mu S_2 \tag{52}$$

Again, using the same procedure as in the complete logistic growth, one can still summarize the system to

$$\frac{dN_1}{dt} = -\tau_1 N_1(K_1 + K_2 - N_1 - N_2) + \mu_1(K_1 - N_1), \tag{53}$$
$$\frac{dN_2}{dt} = -\tau_2 N_2(K_1 + K_2 - N_1 - N_2) + \mu_2(K_2 - N_2). \tag{54}$$

Because $K_i > N_i$, it is clear that the new infection term $\tau_1 N_1(K_1 + K_2 - N_1 - N_2)$ is larger than the non-promiscuous infection term $-\tau_1 N_1(K_1 - N_1)$. In fact, species 1 for example *feels* an infectious carrying capacity of $K_1 + K_2 - N_2 > K_1$. This makes it clear that promiscuity will increase the effective rates of HGT and lead to larger proportions of infected individuals in both species.

To solve this problem analytically and gain further intuition of the system, we can study the symmetric case in which both populations have the same parameters and can share the plasmid. At equilibrium, let us define the total population of both species as $T^* = N_1^* + N_2^*$. Then the equations at equilibrium ($dN_i/dt = 0$) become:

$$\begin{aligned} \tau N_1^*(K_1 + K_2 - T^*) = \mu(K_1 - N_1^*) \\ \tau N_2^*(K_1 + K_2 - T^*) = \mu(K_2 - N_2^*) \end{aligned} \tag{55}$$

We can divide the two equations to eliminate the shared term $(K_1 + K_2 - T^*)$:

$$\frac{N_1^*}{N_2^*} = \frac{K_1 - N_1^*}{K_2 - N_2^*} \tag{56}$$

Cross-multiplying:

$$N_1^*(K_2 - N_2^*) = N_2^*(K_1 - N_1^*) \tag{57}$$

This gives a nonlinear relationship between N_1^* and N_2^*. Along with the constraint $T^* = N_1^* + N_2^*$, this system can be solved numerically or symbolically for specific parameter values.

Symmetric case:

Assume $K_1 = K_2 = K$. By symmetry, we expect $N_1^* = N_2^* = N^*$. Then $T^* = 2N^*$, and the equilibrium equation becomes:

$$0 = -\tau N^*(2K - 2N^*) + \mu(K - N^*) \quad (58)$$

Simplifying:

$$0 = -2\tau N^*(K - N^*) + \mu(K - N^*) \quad (59)$$

If $K \neq N^*$, divide both sides by $K - N^*$:

$$0 = -2\tau N^* + \mu \Rightarrow N^* = \frac{\mu}{2\tau} \quad (60)$$

Thus, in the symmetric case:

$$N_1^* = N_2^* = \frac{\mu}{2\tau}, \quad S_1^* = S_2^* = K - \frac{\mu}{2\tau}. \quad (61)$$

This result, albeit simple, provides the intuition that we needed: if two symmetric populations cannot share a plasmid, they will each have $S_i^* = K - \frac{\mu}{\tau}$ individuals carrying the plasmid. Yet, once promiscuity is possible, and the plasmid can be shared, the effective HGT rate doubles due to the presence of the infected individuals from the other species, and the infected population becomes $S_i^* = K - \frac{\mu}{2\tau}$. In a system where σ species could share the plasmid, one would expect $S_i^* = K - \frac{\mu}{\sigma\tau}$, leading to a system where most individuals in each species carry the plasmid thanks to cross-infection.

Asymmetric case (simulations):
It could be that the asymmetric can be studied analyticaly. If possible, it will yield mostly tedious developments. In any case, we have already obtained the central intuition and so one could simply show the results with simulations, for example:

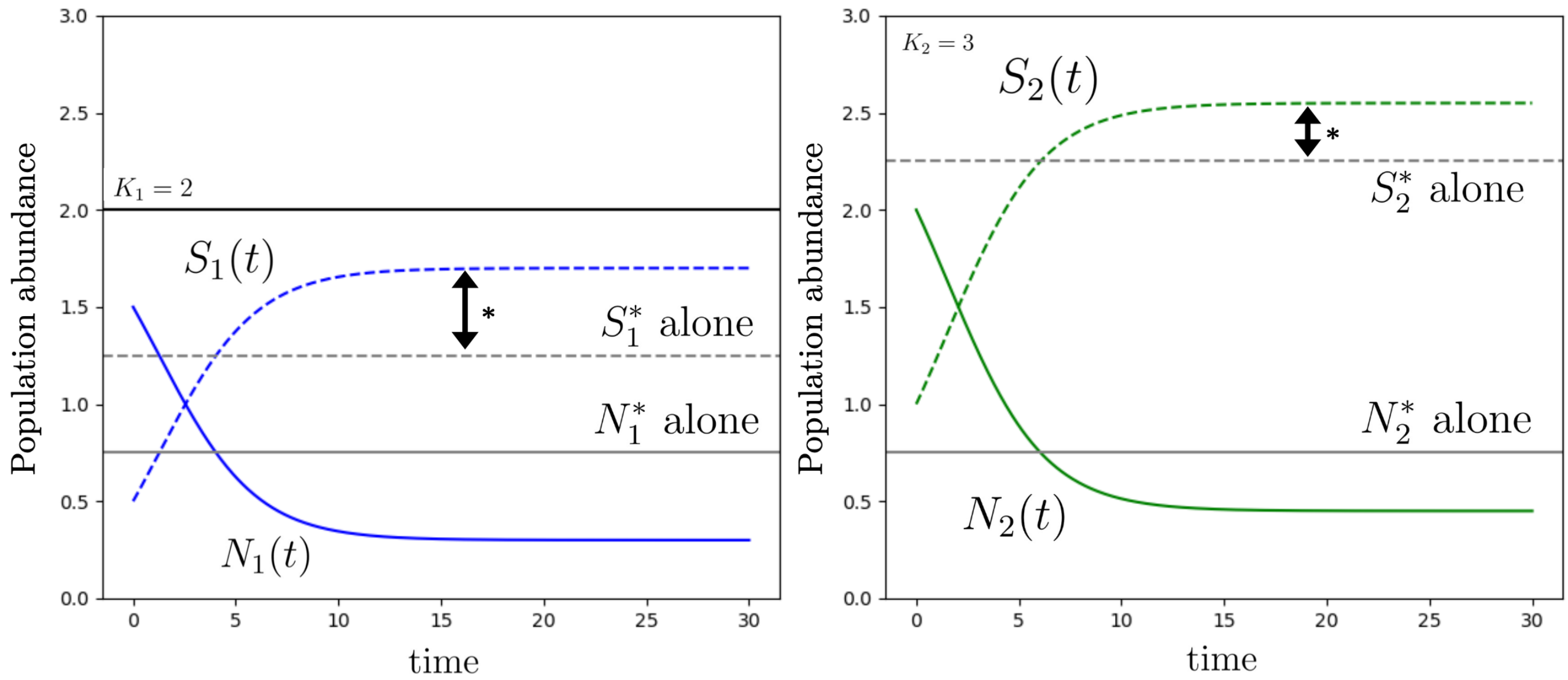

* Promiscuity increases the number of infected individuals, with smaller populations (smaller K, in blue) feeling the largest effect of infections from larger populations (larger K, in green).

The difference with classical SIS models

The classical SIS (Susceptible-Infected-Susceptible) model in epidemiology assumes frequency-dependent transmission, suitable for human populations with a fixed total population size N and a relatively constant number of social contacts. The standard form is:

$$\frac{dI}{dt} = \beta\frac{SI}{N} - \gamma I, \tag{62}$$
$$\frac{dS}{dt} = -\beta\frac{SI}{N} + \gamma I, \tag{63}$$

where S and I are the numbers of susceptible and infected individuals, β is the transmission rate, and γ is the recovery rate. The term $\frac{SI}{N}$ reflects the probability of a contact being with an infected individual (i.e., a frequency-dependent assumption). This is a central difference with our model, that emerges only when we go beyond $N = 1$. These particularities are what was bothering me: because I wanted to introduce two species with different carrying capacities, I had to avoid setting $N = 1$ or $N_i + S_i = 1$, and so the following issues of frequency- vs abundance-dependent infections emerged.

In the classical SIS model, the stable state with susceptible and infected individuals present is $S^* = N\frac{\gamma}{\beta}$ and $I^* = N(1 - \frac{\gamma}{\beta})$. This result is different from the one in our model: now both populations are a function (and fraction) of the total population size. These particularities are what was bothering me, and I was unsure of whether we should incorporate some notion of total population in the main model.

The point here is that, in human populations, the number of social contacts is relatively low compared to the total population. In this context, an infection rate of βSI would not be realistic beyond a certain point, in the sense that, even if the infected population increases a lot, it would not mean that a person's infection rate can increase as much. Rather, the model explores the *probability* that each of our social contacts is with an infected individual, wich is related to the fraction of infections $\frac{I}{N}$.

For bacterial populations undergoing horizontal gene transfer (HGT), such as plasmid exchange, it is more appropriate to use abundance-dependent interactions. In this context, the dynamics can be modeled as described above: the more infected individuals in a given spatial domain, the more likely it is to obtain the promiscuous plasmid (become infected). Bacteria interact through e.g. direct contact or environmental DNA uptake, both of which depend on actual cell abundance rather than relative frequencies. Therefore, using abundance-dependent terms as you originally proposed in the main model better captures the biology of horizontal gene transfer in microbial systems.

High-Dimensional Model Analysis

Community Structure

A key question in our multispecies model is how an engineered plasmid alters community structure. Although HGT raises concerns about unpredictable ecological shifts, our results indicate a robust community response. Structural change is quantified using the Kullback-Leibler (KL) divergence, which measures differences between the pre- and post-inoculation abundance distributions. Across simulations, KL divergence remains low, showing that, even though individual biomasses or strain identities may shift, the overall community composition and coexistence attractor remain stable. Strains are replaced to some degree by their plasmid-bearing counterparts rather than reorganizing the community. This stability persists across parameter regimes, with coexistence between wild-type and synthetic strains remaining the predominant outcome.

BOX 1. Kullback-Leibler Distance

A measure of the changes in population distributions between T_1 and T_2 is needed. Quantifying how much one probability distribution deviates from another allows us to assess shifts in specific species abundance within the community. The Kullback-Leibler (KL) distance helps evaluate the impact of interventions by highlighting differences in species composition. A high KL divergence signals significant changes, while a low value indicates invariance. To calculate the Kullback-Leibler (KL) distance, we first extract the final species abundances from the two different system states we are comparing, at times T_1 and T_2. Each state consists of wild-type and synthetic species, which are summed to obtain total species abundances for each state. Next, we sort the species by their abundances in descending order to ensure proper correspondence between species across the two states. Once sorted, we normalize the abundances by dividing each species abundance by the total sum of abundances in its respective state, transforming them into probability distributions P_{T_1} (for state 1) and P_{T_2} (for state 2). Since the KL divergence involves a logarithm function, we introduce a small constant $\epsilon = 1 \times 10^{-12}$ to prevent numerical errors when probabilities are zero. Finally, we compute the KL divergence using:

$$D_{KL}(P_{T_1} \parallel P_{T_2}) = \sum_i P_{T_1}(i) \log\left\{\frac{P_{T_1}(i) + \epsilon}{P_{T_2}(i) + \epsilon}\right\} \tag{64}$$

In Fig. 1 Kullback-Leibler distances are plotted for a sweep of plasmid transmissibility and loss rate, $\tau \in [0, 0.15]$ and $\mu \in [0, 0.3]$. For each (τ, μ) pair, 200 independent replicas were run with randomized parameters $\rho_k \in [0.8, 1]$, $\beta_k \in [0.7, 0.9]$, $\gamma = 0.3$, $\eta_i \in [0.5, 1]$, $\sigma_i \in [0.5, 0.7]$ and $\epsilon = 10^{-5}$. The resource affected by S are determined by δ_i, see methods in the main text.

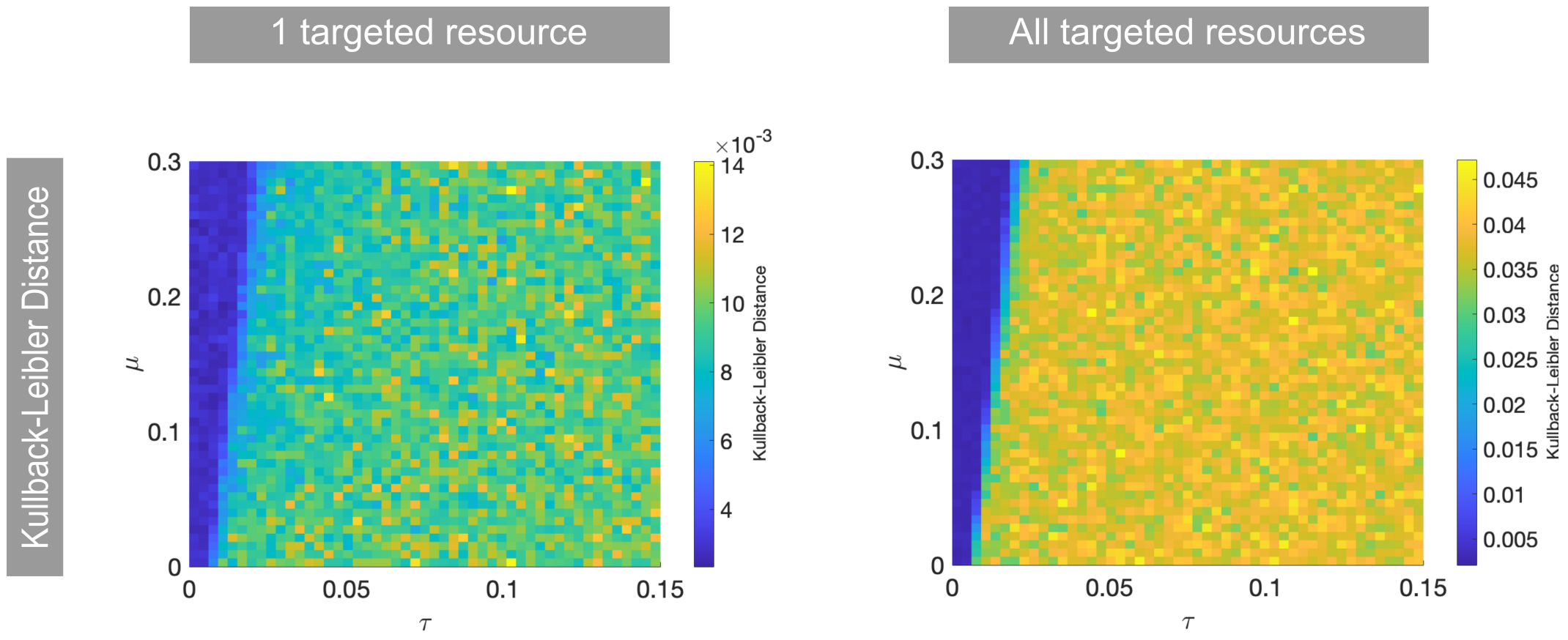

FIG. 1: Kullback-Leibler distances across τ and μ values, before to post-HGT. a) Kullback-Leibler distances when 1 resource of the pool is affected. b) when all resources in the pool are affected.

At the extremes, $\tau = 0$ prevents plasmid spread and only wild types persist, while $\mu = 0$ prevents plasmid loss and synthetic variants fully dominate (see main text). Between these limits, varying degrees of coexistence arise. We ask whether this coexistence increases strain-level biodiversity. If m^* denotes the number of wild-type species surviving before inoculation, then post-HGT biodiversity is always at least m^* and can reach $2m^*$, meaning all species may persist in both wild-type and synthetic forms (Fig.4c, main text and Fig. 2a here in the SM). In the limiting cases, the community contains either m^* wild types or m^* synthetic variants, but intermediate regimes consistently yield more than m^* strains. At high τ, all synthetic strains persist while only the most abundant wild types remain; at intermediate τ, diversity is maximal, with all species maintaining both wild-type and plasmid-bearing forms. Thus, effective HGT does not require high τ: intermediate transmissibility maximizes coexistence and strain diversity.

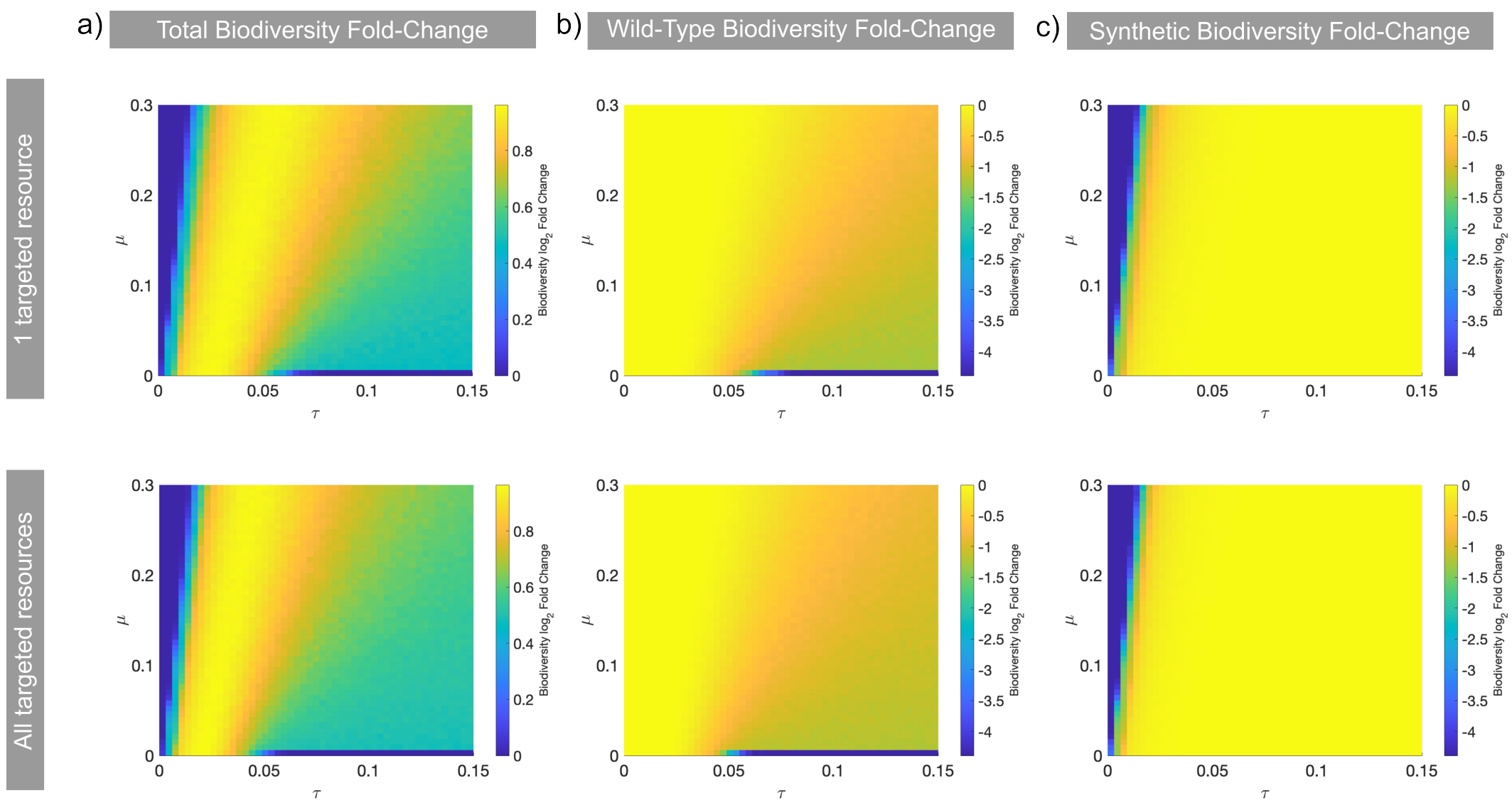

FIG. 2: Biodiversity fold-change across τ and μ values, before and post-HGT. a) Total biodiversity fold-change, synthetic plus wild-types. b) Wild-type diversity fold-change. c) Synthetic diversity fold-change

In Fig. 2, we show the biodiversity fold change when either one or all resources are targeted by the synthetic strains, see methods in the main text. As stated in the main text, coexistence and diversity metrics are insensitive to which resources are affected. Wild-type biodiversity decreases at high transmissivities (Fig. 2b), and these losses

map directly onto their synthetic counterparts (Fig. 2c). This phenomenon is consistent with the predictions from the section above, "Plasmid transfer in a simple two-species model: Epidemiologic Approach".

Varying the Number of Targeted Resources

In the main text, we examined the ecological consequences of horizontal gene transfer under the scenario in which only one resource is affected by the plasmid-encoded damping function. As defined by

$$F(R_k, S_i) = \frac{\beta_k R_k}{1 + \delta_{k\alpha} \frac{\sum_{i=1}^{n} S_i}{\lambda}}, \tag{65}$$

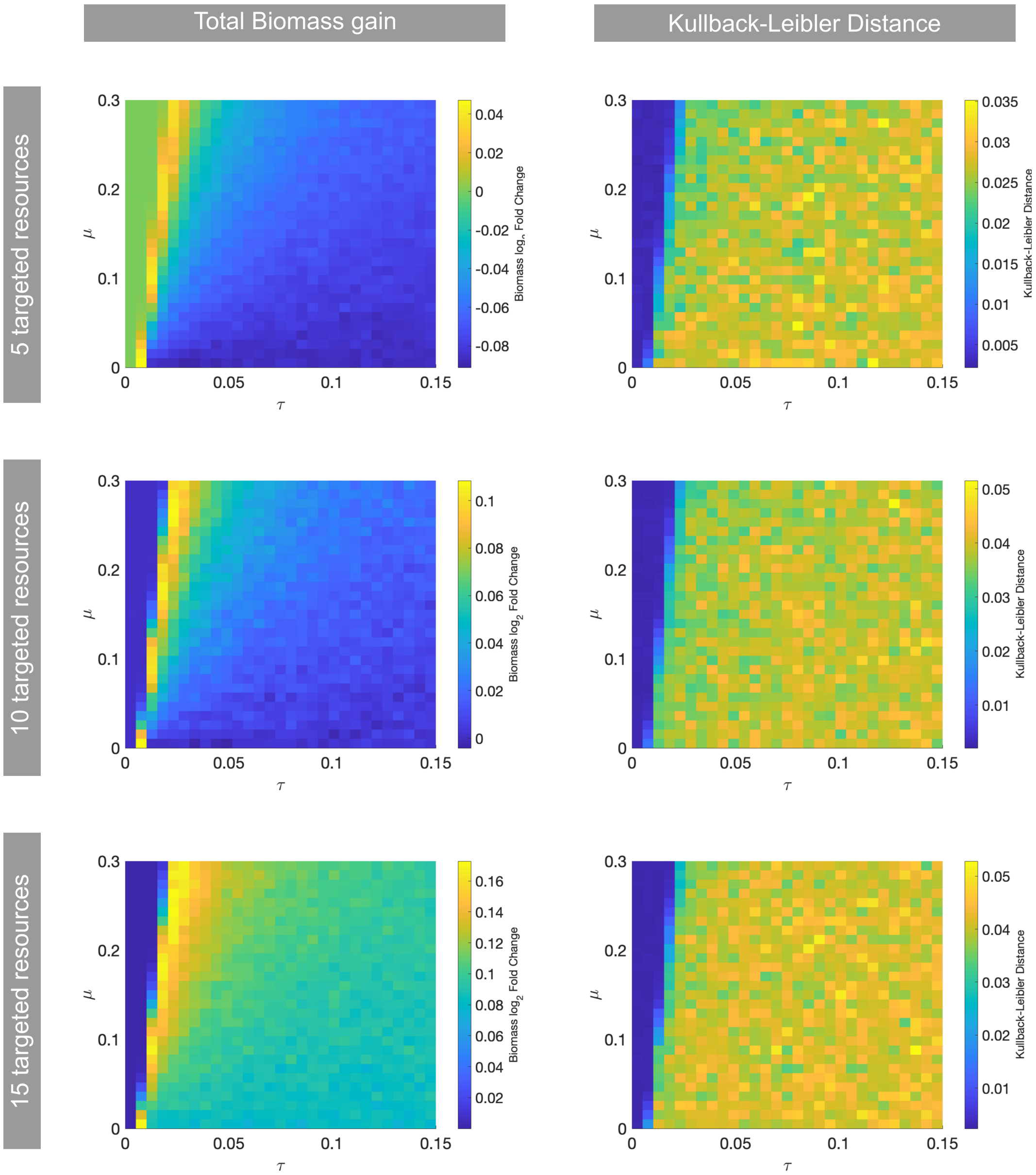

FIG. 3: Biomass fold-change and Kullback-Leibler distances across τ and μ values, before and post-HGT, when 5, 10 or 15 resources are affected by the synthetic presence in the system.

the presence of synthetic species introduces a non-linear inhibition of the decay rate of resource R_k. The indicator $\delta_{k\alpha}$ selects the subset of resources targeted by the engineered function, with $\delta_{k\alpha} = 1$ if resource k is affected and $\delta_{k\alpha} = 0$ otherwise. Thus, the engineered damping may apply to a single resource or to any subset of the full resource pool $\{R_k\}_{k=1}^{m}$, up to and including all resources.

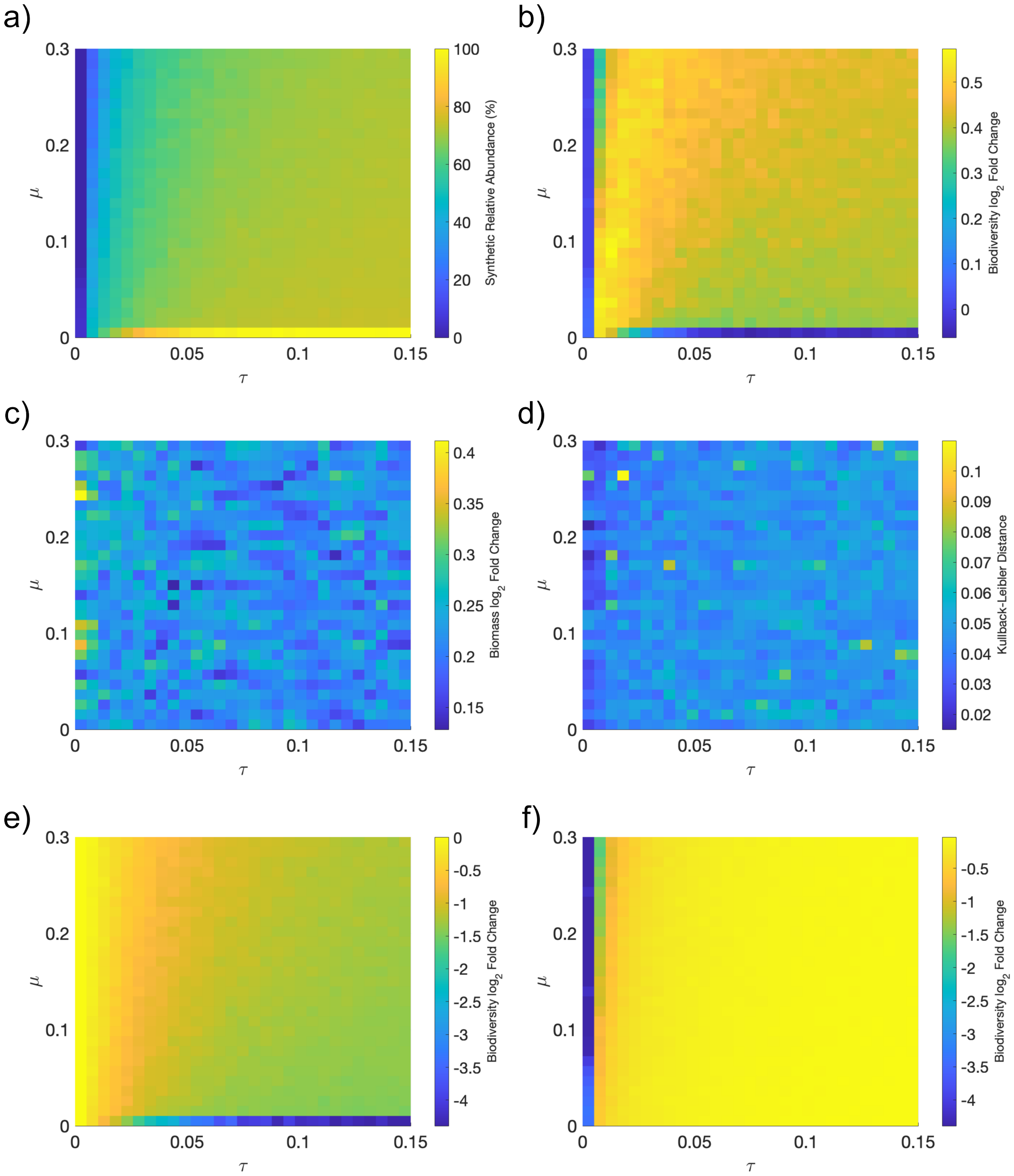

FIG. 4: Statistical analysis of the high-dimensional model for $\eta' = \eta - 0.1\eta$ for a community with $n = m = 20$. The number of targeted resources is an intermediate value of 10. After the first inoculation of the synthetic plasmid carrier at $t = T_1$, a new stability state is reached at $t = T_2$. In (a), the relative abundance of S_i vs N_i, in (b) the biodiversity fold change, in c) the biomass fold change, in d) the Kullback–Leibler distance, in e) wild-type diversity fold-change and in f) synthetic diversity fold-change across τ and μ values are shown. The results remain consistent across parameters, here with fully randomized values: $\rho_k \in [0, 1]$, $\beta_k \in [0, 1]$, $\gamma = 0.3$, $\eta_i \in [0, 1]$, $\eta' = \eta - 0.1\eta$ and $\delta_i \in [0, 1]$.

To examine how the scope of the engineered intervention influences ecosystem dynamics, we extend the single-resource and full-resource analyses from the main text to intermediate cases in which the plasmid affects 5, 10, or 15 available resources. For each configuration, we performed the full (τ, μ) parameter sweep and quantified synthetic relative abundance, biodiversity fold change, biomass fold change, and the Kullback–Leibler divergence (see Box 1) between community states at times T_1 and T_2, averaging over 200 independently randomized ecosystem replicas. As

stated in the main text and earlier in this supplement, synthetic relative abundance and total biodiversity fold change remain independent of how many resources are targeted. In contrast, biomass fold change increases substantially when more resources are affected: broader targeting directly yields larger biomass gains (see main text for the 1-resource and all-resource limits). Results for the intermediate scenarios are shown in Fig. 4. Importantly, the Kullback–Leibler distance remains low across all three cases. Fig. 4, together with Fig. 4d,e in the main text, illustrates how the engineered functionality transitions from a localized perturbation to a broad ecological modification that simultaneously influences multiple limiting factors. As expected from the saturating form of the damping term $F(R_k, S_i)$, increasing the number of damped resources amplifies biomass gains while still preserving coexistence and maintaining low divergence from the original community structure. These supplementary graphs thus allow direct comparison of ecological outcomes as the intervention scales from single-resource modulation to a maximal system-wide effect.

Fully Randomized Parameter Regime

In the main text and in the analyses presented above, we evaluated ecosystem responses under a controlled parametrization in which key ecological and metabolic variables were drawn from restricted intervals: $\rho_k \in [0.8, 1]$, $\beta_k \in [0.7, 0.9]$, $\gamma = 0.3$, $\eta_i \in [0.5, 1]$, $\eta_i' = \eta_i - 0.1\eta_i$, and $\delta_i \in [0.5, 0.7]$. These choices ensured tractability while still allowing substantial heterogeneity across species and resources, yet the results were shown to be robust across all parameter space.

To further test the generality of our conclusions, we performed an additional analysis using *fully randomized* ecological and metabolic parameters, removing all structured constraints. In this extended setup, each simulation draws parameters independently from uniform distributions spanning the entire unit interval:

$$\begin{aligned} &\rho_k \sim U(0,1), \qquad \beta_k \sim U(0,1), \qquad \eta_i \sim U(0,1), \\ &\eta_i' = \eta_i - 0.1\eta_i, \qquad \delta_i \sim U(0,1), \end{aligned}$$

Across this fully randomized parameter ensemble, *all qualitative results remained unchanged.* Coexistence patterns, the stability of biodiversity, the low Kullback–Leibler divergence between pre- and post-intervention communities, and the biomass gains acording to the number of targeted resources. These findings confirm that the ecological behaviors reported in the main text are not artifacts of a particular parametrization, but instead reflect robust, model-inherent features of HGT-mediated ecosystem engineering.
